# Whole-genome analysis revealed the growth-promoting and biological control mechanism of the endophytic bacterial strain *Bacillus halotolerans* Q2H2, with strong antagonistic activity in potato plants

**DOI:** 10.3389/fmicb.2023.1287921

**Published:** 2024-01-03

**Authors:** Yuhu Wang, Zhenqi Sun, Qianqian Zhao, Xiangdong Yang, Yahui Li, Hongyou Zhou, Mingmin Zhao, Hongli Zheng

**Affiliations:** ^1^College of Horticulture and Plant Protection, Inner Mongolia Agricultural University, Hohhot, China; ^2^Institute of Agro-Food Technology, Jilin Academy of Agricultural Sciences, Changchun, China; ^3^Jilin Provincial Key Laboratory of Agricultural Biotechnology, Jilin Academy of Agricultural Sciences, Changchun, China

**Keywords:** bacterial endophytes, *Bacillus halotolerans*, plant growth-promoting, antagonistic activities, biological control, whole-genome sequencing

## Abstract

**Introduction:**

Endophytes are colonizers of healthy plants and they normally exhibit biocontrol activities, such as reducing the occurrence of plant diseases and promoting plant growth. The endophytic bacterium *Bacillus halotolerans* Q2H2 (Q2H2) was isolated from the roots of potato plants and was found to have an antagonistic effect on pathogenic fungi.

**Methods:**

Q2H2 was identified by morphological observations, physiological and biochemical identification, and 16S rRNA gene sequence analysis. Genes related to the anti-fungal and growth-promoting effects were analyzed using whole-genome sequencing and comparative genomic analysis. Finally, we analyzed the growth-promoting and biocontrol activities of Q2H2 in potato plants using pot experiments.

**Results:**

Antagonism and non-volatile substance plate tests showed that Q2H2 had strong antagonism against *Fusarium oxysporum*, *Fusarium commune*, *Fusarium graminearum*, *Fusarium brachygibbosum*, *Rhizoctonia solani* and *Stemphylium solani*. The plate test showed that Q2H2 had the ability to produce proteases, cellulases, β-1,3-glucanase, dissolved organic phosphate, siderophores, indole-3-acetic acid (IAA), ammonia and fix nitrogen. The suitable growth ranges of Q2H2 under different forms of abiotic stress were pH 5–9, a temperature of 15–30°C, and a salt concentration of 1–5%. Though whole-genome sequencing, we obtained sequencing data of approximately 4.16 MB encompassed 4,102 coding sequences. We predicted 10 secondary metabolite gene clusters related to antagonism and growth promotion, including five known products surfactin, bacillaene, fengycin, bacilysin, bacillibactin, and subtilosin A. Average nucleotide identity and comparative genomic analyses revealed that Q2H2 was *Bacillus halotolerans*. Through gene function annotation, we analyzed genes related to antagonism and plant growth promotion in the Q2H2 genome. These included genes involved in phosphate metabolism (*pstB*, *pstA*, *pstC*, and *pstS*), nitrogen fixation (*nifS*, *nifU*, *salA*, and *sufU*), ammonia production (*gudB*, *rocG*, *nasD*, and *nasE*), siderophore production (*fhuC*, *fhuG*, *fhuB*, and *fhuD*), IAA production (*trpABFCDE*), biofilm formation (*tasA*, *bslA*, and *bslB*), and volatile compound production (*alsD*, *ilvABCDEHKY*, *metH*, and *ispE*), and genes encoding hydrolases (*eglS*, *amyE*, *gmuD*, *ganB*, *sleL*, and *ydhD*). The potato pot test showed that Q2H2 had an obvious growth-promoting effect on potato roots and better control of *Fusarium* wilt than carbendazim.

**Conclusion:**

These findings suggest that the strain-specific genes identified in bacterial endophytes may reveal important antagonistic and plant growth-promoting mechanisms.

## 1 Introduction

Bacterial endophytes are a group of microorganisms that live in plants, but do not cause disease (Gouda et al., 2016). They can invade and colonize host plants, directly or indirectly provide nutrients for plant growth, and even help plants resist disease (Pageni et al., 2014). Several studies have shown that endophytes regulate plant growth through nitrogen fixation, phosphate solubilization, siderophore production, 1-aminocyclopropane-1-carboxylate (ACC) deaminase activity, indole-3-acetic acid (IAA) synthesis, and antagonistic effects against pathogens (Yan et al., 2018). Endophytic bacteria with antagonistic activity are widely used for the biological control of plant diseases and can not only reduce the occurrence of diseases, but can also promote plant growth.

Endophytic nitrogen-fixing bacteria invade the plant body and colonize xylem vessels, exerting nitrogen-fixing ability. Fixed nitrogen can be directly absorbed and utilized by plants. These bacteria can also invade most vegetative organs of their host plants and play a role in nitrogen fixation by providing available nitrogen sources for the plants (Wang et al., 2022a). Recent research has indicated that endophytic nitrogen-fixing bacteria do not cause adverse reactions in plants (Pankievicz et al., 2015). After inoculation with the endophytic nitrogen-fixing bacterium *Klebsiella* DX120E, the height, fresh weight, and chlorophyll content of sugarcane significantly increases. DX120E colonization has been observed in root hairs, new lateral roots, wounds, and leaves using green fluorescent protein labeling (Gu et al., 2018). *Azorhizobium caulinodans* invades the roots of grains through intercellular interactions between epidermal cells and it colonizes various parts of plants, including the xylem (Dreyfus et al., 1988). Many endophytic bacteria have successfully colonized non-leguminous plants, such as rice, wheat, corn, and sugarcane, forming symbiotic relationships and fixing nitrogen (Santoyo et al., 2016).

Phosphate-solubilizing bacteria can dissolve insoluble phosphates or organic phosphorus compounds. They participate in the material cycle of soil ecosystems and indirectly promote plant growth and development (Li et al., 2021). The emergence of phosphate-solubilizing bacteria increases the soil phosphate content, which is beneficial for plants to absorb phosphorus and it improves plant nutrition (Liu et al., 2023). Therefore, endophytic bacteria that can dissolve phosphates are expected to promote sustainable agriculture. *Bacillus subtilis* RS10, *Enterobacter roggenkampii* ED5, and *B. subtilis* EA-CB0575 have been reported to solubilize phosphate and promote plant growth (Franco-Sierra et al., 2020; Guo et al., 2020; Iqbal et al., 2021).

Endophytic bacteria secrete plant hormones such as IAA, gibberellins, and cytokinins that stimulate plant growth (Wang et al., 2022a). Among these bacteria, the IAA-producing strains have been extensively studied. IAA promotes cell elongation and growth and seed germination and it is one of the earliest and most extensively studied plant growth hormones. IAA synthesis plays a crucial role in the promotion of plant growth (Santoyo et al., 2016). *B. subtilis* LK14 produces IAA and ACC deaminases *in vitro*. After the inoculation of tomato plants with endophytic *B. subtilis* LK14, the biomass and chlorophyll content of the stems and roots significantly increases (Khan et al., 2016). Endophytic *Burkholderia* PsJN produces ACC deaminase and promotes plant growth (Mosqueda et al., 2020). Mutants that lose their ACC deaminase activity no longer promote root elongation in rapeseed seedlings (Sun et al., 2009).

Siderophores are small-molecule substances produced by microorganisms under low-iron conditions. They have a high affinity for Fe^3+^ and can chelate iron from the environment, thereby providing iron to microorganisms. Siderophores usually combine with iron to form complexes, contributing to iron uptake directly from the complexes or by ligand exchange (Garzón-Posse et al., 2019). As bacterial siderophores increase iron absorption, they also increase plant growth and development. For example, siderophore-producing microorganisms are found in rice, wheat, corn, peanut, and other crops (Santoyo et al., 2016; Afzal et al., 2019; Deng et al., 2023). Siderophores can form steady complexes with heavy metals, such as Cd, Cu, Pb, Zn, or radionuclide U, thereby reducing phytotoxicity in heavy-metal-contaminated areas and improving plant growth (Grobelak and Hiller, 2017).

Previous studies have shown that endophytic antagonistic antibacterial agents can stabilize colonization in plants, and the production of chitinases, proteases, and antibacterial peptides can destroy foreign pathogenic bacteria, thereby protecting plants from disease. Antibacterial substances are secreted by antagonistic bacteria that inhibit the growth of plant pathogens to achieve biological control (Boro et al., 2022). There are two main types of antibacterial substances secreted by antagonistic bacteria: small-molecule antibiotics and cell wall hydrolases or antibacterial proteins with large molecular weights. Among the most commonly used antagonistic microorganisms, *Bacillus* is a prominent genus used as a biocontrol agent in crop production, as this genus shows strong stress resistance and good environmental adaptability. Fengycin, iturin A, and surfactin produced by *Bacillus amyloliquefaciens* PPCB004, and bacillomycin, fengycin, and iturin A produced by *B. subtilis* UMAF6614 and UMAF6639 are key factors in antagonism against fungal pathogens (Arrebola et al., 2010; Zeriouh et al., 2011; Bonaterra et al., 2022). The endophyte *Bacillus velezensis* BR-01 shows strong antagonistic activity against a variety of rice pathogens and significantly controls rice bacterial blight, because of its production of antibacterial peptides, such as surfactin, fengycin, and iturin, and antagonism-related enzymes, such as cellulase and β-1,3-glucanase (Zhou et al., 2022). *Bacillus halotolerans* QTH8 inhibits the mycelial growth of various pathogenic fungi, such as *Fusarium pseudograminearum*, and wheat plants treated with QTH8 have a significantly reduced occurrence of wheat crown rot (Li et al., 2022).

In addition to promoting plant growth, endophytes can enhance the tolerance of host plants to abiotic stress. Drought stress significantly affects the normal growth, development, and reproduction of crops. Chandra et al. (2019) showed that mixed inoculation of wheat with plant growth-promoting bacteria (PGPB) effectively promotes plant growth; significantly increases the leaf nutrient content, especially under conditions of water stress; and significantly improves the antioxidant capacity of the wheat plants. Under salt stress, PGPB participates in regulating the expression of related genes in plants, thereby enhancing their adaptability to saline environments (Li X. X. et al., 2016). Corn and potato plants inoculated with PGPB enhance their adaptability to salt stress by regulating the absorption of Na^+^ and K^+^ (Rojas-Tapias et al., 2012; Tahir et al., 2019).

Whole-genome analysis of endophytes can be used to classify genes associated with antagonistic and plant-growth-promoting activities, while providing insights into their molecular and functional mechanisms (Franco-Sierra et al., 2020). A variety of plant-growth-promoting genes have been identified in endophytic bacteria (Coulson and Patten, 2015; Asaf et al., 2018). These genes include those encoding nitrogen fixation, ACC deaminase activity, siderophore biosynthesis, plant hormone (IAA, acetyl, and 2,3-butanediol) production, and the synthesis of antibacterial compounds (Iqbal et al., 2021). The whole genome of *E. roggenkampii* ED5 has been sequenced, and a series of genes related to plant-growth-promotion (PGP) activity have been identified. This is the first genome sequence of *E. roggenkampii*, and genes related to nitrogen fixation, plant hormone production, ACC deaminase, biotic and abiotic stress, resistance induction, and root colonization have been identified (Guo et al., 2020). Based on whole-genome sequencing data for *B. subtilis* EA-CB0575, genes involved in indole, siderophore, lipopeptide, volatile compound, bacitracin, and nitrogenase production have been predicted (Franco-Sierra et al., 2020). Whole-genome sequencing of endophytic *Bacillus toyonensis* BAC3151 revealed its potential role in anti-microbial development associated with the control of crop diseases after analyzing secondary metabolites (Lopes et al., 2017). Whole-genome sequencing can be used to obtain the complete sequences of bacterial genomes, allowing a comprehensive understanding of bacterial genome composition and structure. The growth-promoting and antagonistic mechanisms of bacteria can be analyzed at the genomic level. This provides valuable bacterial genome information and excellent bacterial species resources.

We isolated an endophytic bacterial strain, Q2H2, with strong antagonistic activity against potato pathogenic fungi at the early stage of infection. The purpose of this study is to investigate the PGP characteristics and biological control activity of Q2H2. Whole-genome sequencing of Q2H2 to further explore genes related to its growth-promoting and biocontrol activities, which may help explore the possible antagonistic mechanism of Q2H2. Analysis of the genomic characteristics of Q2H2 and gene clusters that possibly related to antibacterial peptides, hydrolases, phosphate solubilization, nitrogen fixation, siderophore production, and IAA production were performed. Our findings provide fundamental knowledge of the endophytic strain Q2H2 and the possible antagonistic and PGP mechanisms it employs, which will be helpful for its potential future applications in enhancing the disease resistance of potatoes.

## 2 Materials and methods

### 2.1 Microorganisms and culture conditions

The endophytic strain Q2H2 was isolated from potato roots (Atlantic variety) and stored at −80°C in 50% glycerol. The cells were cultured in Luria-Bertani (LB) medium, as described by Wang et al. (2022b).

Genomic DNA was extracted from an overnight LB liquid cell suspension of the endophytic strain Q2H2 using a Bacterial Genomic DNA Extraction Kit (TIANGEN BIOTECH, Beijing, China). DNA quality and concentration were estimated using a Nanodrop ONE instrument (Thermo Fisher Scientific, Waltham, MA, USA). The 16S rRNA gene of Q2H2 was amplified by polymerase chain reaction (PCR) using primers 7F and 1540R (Xiu et al., 2022). The PCR system and program were operated according to the Taq polymerase instructions (TaKaRa, Dalian, China). Genomic DNA was used as the template for PCR amplification (Table 1; Wang et al., 2022b). The PCR products were purified and sequenced by BGI (Shenzhen, China). The sequencing results were compared online with those from the National Center for Biotechnology Information (NCBI) GenBank database. A phylogenetic tree was created using Molecular Evolutionary Genetic Analysis (MEGA7) software (version 7.0, Mega Limited, Auckland, New Zealand).

**Table 1:** 16S rRNA PCR amplification experiments conditions.

16S rRNA gene PCR amplification experiments conditions	
Product size	About 1600 bp
PCR primer	7F 5′-CAGAGTTTGATCCTGGCT-3′ 1540R 5′-AGGAGGTGATCCAGCCGCA3′
PCR conditions	Initial temperature (98°C for 3 min), start cycles (29), Denaturation (98°C for 10s), Annealing (50°C for 30s), Elongation (72°C for 1 min 50 s), Final extensions (72°C for 10 min).

### 2.2 Physiological and biochemical tests

Physiological and biochemical characterization tests, such as Gram staining, catalase enzyme assay, citrate utilization, malonate utilization, methyl red, oxidase, Voges–Proskauer (V-P), gelatin liquefaction, starch hydrolysis, and fat hydrolysis tests were performed on Q2H2. These physiological and biochemical tests were performed in triplicate.

### 2.3 Antagonism assay against pathogenic fungi

*In vitro* antifungal activity tests were performed for strain Q2H2 using the following six pathogenic fungi: *Fusarium oxysporum, Fusarium commune, Fusarium graminearum, Fusarium brachygibbosum, Rhizoctonia solani*, and *Stemphylium solani*. The two-point confrontation method was used on potato dextrose agar (PDA) medium. 48 h old pathogenic fungi with a diameter of 5 mm were cut from the PDA plates using a perforator. 24 h old bacterial Q2H2 colonies and pathogenic fungi were inoculated 2 cm from the center of the PDA plate. The plate containing only pathogenic bacteria was used as the control, and was cultured at 28°C until the fungus grew to cover approximately 3/4 of the dish diameter. The diameter of the pathogen was measured using the cross-measurement method (Yang et al., 2017), and the antifungal rate of the strain was calculated according to Equation 1.


(1) $$R_{1}\left(\%\right)=\frac{r_{0}-r_{1}}{r_{0}-r}\times 100\%$$

where, *R_1_* is the antifungal rate, *r_0_* is the radius of the control group, *r_1_* is the radius of the treatment group, and *r* is the radius of the punch.

Non-volatile substance antibacterial test: A single colony of strain Q2H2 was selected and incubated in nutrient broth (NB; 10 g of peptone, 3 g of beef extract, 5 g of NaCl, 1,000 mL of distilled water; pH 7.0–7.2) at 28°C and 180 rpm for 24 h to prepare the fermentation broth. The fermentation broth was diluted to an optical density at 600 nm (OD_600_) of 1 and centrifuged at 12,000 rpm for 15 min at room temperature. The resulting supernatant was filtered through a 0.22 μm filter to remove the bacteria. Two hundred milliliters of PDA culture medium was heated until it melted, and after cooling to approximately 50°C, 20 mL of filtered supernatant was added and mixed well to prepare a culture medium plate. After the plate solidified, it was inoculated with *F. oxysporum* and the five other pathogenic fungal cakes. A PDA plate without the fermentation supernatant was used as a control, with three replicates for each treatment. After 5 days of cultivation at 28°C, the colony growth diameter was measured and the inhibition rate was calculated using Equation 2 (Xu et al., 2020).


(1) $$R_{2}\left(\%\right)=\frac{d_{0}-d_{1}}{d_{0}-d}\times 100\%$$

where *R*_2_ is the antifungal rate, *d_0_* is the diameter of the control group, *d_1_* is the diameter of the treatment group, and *d* is the diameter of the punch.

Volatile substance antibacterial test: Strain Q2H2 was cultured on nutrient agar (NA) medium (10 g of peptone, 3 g of beef extract, 5 g of NaCl, 18–20 g of agar, and 1,000 mL of distilled water; pH 7.0–7.2). The 5 mm fungal cake of 48 h old pathogen was placed in the center of another plate on PDA medium. The culture dishes containing the bacteria and pathogens were then placed face to face, sealed with sealing film, and incubated at 28°C for 5 days. A plate containing nonbacterial NA culture medium was used as a control.

### 2.4 Q2H2 abiotic stress test *in vitro*

The growth of Q2H2 under different temperatures (10–45°C), pH values (4–11), and salt concentrations (1–11%) was measured to determine its ability to withstand three abiotic stresses. A spectrophotometer was used to determine the growth in LB broth by measuring the absorbance at 600 nm (Guo et al., 2020).

#### 2.4.1 Temperature tolerance

For the temperature tolerance test, 0.2 mL of a Q2H2 bacterial suspension cultured overnight was added to 20 mL of LB broth and cultured at 10, 15, 20, 25, 30, 35, 40, and 45°C at 180 rpm for 24 h. The OD_600_ value was then determined.

#### 2.4.2 pH tolerance

The pH tolerance of Q2H2 was assessed using LB broth adjusted to pH values of 4, 5, 6, 7, 8, 9, 10, and 11 using sterile buffers. A 0.2 mL sample of fresh bacterial suspension was transferred to 20 mL of LB broth with different pH values, and incubated in a vibration incubator at 28°C and 180 rpm for 24 h. The OD_600_ value was then determined.

#### 2.4.3 Salinity tolerance

For the salinity tolerance test, 20 mL of LB broth was supplemented with 1, 3, 5, 7, 9, or 11% NaCl. A 0.2 mL sample of bacterial suspension was inoculated into the LB broth tubes and incubated at 28°C and 180 rpm in a shaker incubator for 24 h. Growth was calculated by determining the OD_600_ value.

### 2.5 Detection of antagonism-related lytic enzymes and PGP activities

The protease, cellulase, glucanase, and chitinase activities of Q2H2 have previously been detected on agar plates containing skim milk powder, sodium carboxymethyl cellulose, β-glucan, and colloidal chitin, respectively (Zhou et al., 2022). A toothpick was used to pick single colonies of Q2H2 cultured overnight. The colonies were inoculated on four types of test media and cultured at 28°C for 2–4 days. The ability of the antagonistic bacteria to produce the four types of hydrolytic enzymes was determined by observing the size of the hydrolytic halo around the colony.

The potential of Q2H2 to solubilize phosphate was qualitatively evaluated using an inorganic phosphate culture medium and an organic phosphate medium with a fresh egg yolk solution (Gao et al., 2015).

Nitrogen-fixing ability was measured using Ashby’s nitrogen-free medium (Singh et al., 2020). Q2H2 was inoculated into Ashby’s medium and cultured at 28°C for 7 days to determine its viability.

Q2H2 was incubated in 10% sterile peptone water at 28°C for 72 h and the development of a yellow color after the addition of Nessler’s reagent (0.5 mL) was used to confirm ammonia production (Guo et al., 2020).

Chrome azurol S (CAS) medium was used to determine the ability of the antagonistic bacteria to produce siderophores. Q2H2 was cultured in solid LB medium, and a single colony was picked with a sterile toothpick, inoculated on the CAS detection medium, and cultured at 28°C for 2–4 days. A yellow or brown halo around the colonies was attributed to the presence of siderophores (Schwyn and Neilands, 1987).

IAA production was analyzed using the Salkowski colorimetric method in the presence of tryptophan (Patten and Glick, 2002). Strains were grown overnight in Dworkin and Foster (DF) medium (4 g of KH_2_PO_4_, 6 g of Na_2_HPO_4_, 0.2 g of MgSO_4_·7H_2_O, 2 g of (NH_4_)_2_SO_4_, 2 g of glucose, 2 g of sodium gluconate, 2 g of citric acid, 0.1 mL each of component 1 and component 2 solutions, and 1,000 mL of distilled water; pH 7.2). Component 1 was prepared by dissolving 11.19 mg of MnSO_4_·H_2_O, 78.22 mg of CuSO_4_·5H_2_O, 124.6 mg of ZnSO_4_·7H_2_O, 10 mg of H_3_BO_3_, and 10 mg of MoO_3_ in 100 mL of sterile water. Component 2 was prepared by dissolving 100 mg of FeSO_4_·7H_2_O in 10 mL of sterile water (Zhang et al., 2016). Two hundred microliters of the culture was transferred to DF medium containing 0.1% L-tryptophan. The strains were cultured at 28°C for 7 days and then centrifuged at 8,000 rpm for 10 min. One milliliter of the fermentation supernatant was mixed with 2 mL of Fe-H_2_SO_4_ solution (1 mL of 0.5 M FeCl_3_·6H_2_O in 75 mL of 6.13 M H_2_SO_4_) and placed in a dark room for 45 min. The IAA concentration was determined by measuring the absorbance of the samples at 450 nm (Amprayn et al., 2012).

### 2.6 Genome sequencing and functional annotation

The sample preparation and genome sequencing methods have previously been described by Wang et al. (2022b). Q2H2 samples were sent to Biomarker Technologies (Beijing, China) for genome sequencing using single-molecule real-time sequencing on the PacBio platform.

Genome component analysis and functional annotation of Q2H2 were performed using a method described by Wang et al. (2022b).

Genes related to phosphate solubilization, nitrogen fixation, IAA synthesis, and hydrolase were analyzed using the Kyoto Encyclopedia of Genes and Genomes (KEGG) database. Gene clusters of the secondary metabolites of Q2H2 were investigated.

### 2.7 Comparative genomics analysis of Q2H2

Average nucleotide identity (ANI) analysis was performed as described by Wang et al. (2022b). The ANI values of Q2H2 compared with eight closely related strains were calculated using the JspeciesWS online tool (Richter et al., 2016), and the results are presented as heat maps (Chen et al., 2020).

Based on the results of the ANI analysis of the Q2H2 genome, the two strains with the closest genetic relationship to Q2H2, *Bacillus halotolerans* strain ZB201702 (ZB201702) and *B. subtilis* subsp. *spizizenii* ATCC 6633 (ATCC 6633) were used for comparative genomic analysis. The genome sequences of *B. halotolerans* ZB201702 (GenBank accession number: NZ_CP029364.1) and ATCC 6633 (GenBank accession number: NZ_CP034943.1) were downloaded from the NCBI database. OrthoMCL Version 2.0 software (Li et al., 2003) was used to perform family clustering of the protein sequences predicted by the sequenced strains and protein sequences of the reference genome. We analyzed the gene families, including strain-specific and common gene families, in different strains. Venn diagrams or petal diagrams were constructed to identify gene families. Functional annotations based on the Pfam database were used. For genome collinearity analysis using the genomes of ZB201702 and ATCC 6633 as reference genomes, the protein sequences of Q2H2 were compared with those of the reference genomes using BLAST. Based on the position of the homologous genes in the genome sequence, a collinear relationship was obtained at the nucleotide level.

### 2.8 Growth-promotion and biological control of potted potato plants

The potato plant growth-promotion test methods were based on those described by Wang et al. (2022b). Q2H2 was streaked on NA medium until colonies grew. Q2H2 colonies were picked, inoculated into 100 mL of liquid NB medium, and cultured at 28°C and 180 rpm for 24 h. The resultant liquid was centrifuged at 4,000 rpm for 10 min, suspended in an equal volume of sterile water, diluted to an OD_600_ of 1, and set aside. In this study, we used the Atlantic potato variety. Soil and nutrient-rich soil were mixed at a ratio of 1:1. A sprouted potato tuber was sown in each flowerpot, and when the potato had 4–5 leaves, it was inoculated with 50 mL of the above bacterial suspension, or 50 mL of sterile water as a negative control, using the root irrigation method. Each treatment was inoculated with three pots of potato plants. The plants were grown under greenhouse conditions (22°C, 16 h of light, 8 h of dark). To detect the effect of Q2H2 on plant growth, we measured the plant height, fresh weight, root weight, and chlorophyll and nitrogen content of the plants 30 days after inoculation. This experiment was performed in triplicate.

A single colony of strain Q2H2 was selected, inoculated into NB medium and cultivated at 28°C with shaking until reaching an OD_600_ of 1. Potato tubers were used as seed potatoes. They were sown in flower pots, with one per pot, and cultivated at 22°C under greenhouse conditions. A *F. oxysporum* cake was inoculated onto potato dextrose broth, oscillated at 28°C and 180 rpm for 7 days, and filtered to remove the hyphae. The concentration of the spore suspension was adjusted to 10^7^ spores/mL and then reserved. When the potato plant had 4–5 leaves, 50 mL of the prepared Q2H2 bacterial fermentation broth was poured randomly into the soil around the base of the plant. After 24 h, 50 mL of the *F. oxysporum* spore suspension was randomly poured into the potato pot by needling through the stem base. After inoculation with 50 mL of 50% carbendazim wettable powder (diluted 1:1,500) for 24 h, *F. oxysporum* was inoculated as a chemical control. Potato plants inoculated with only *F. oxysporum* were used as a positive control. Only 50 mL NB medium was watered as a blank control. Each treatment was inoculated with three pots of potato plants. When the positive control showed obvious symptoms of potato wilt disease, an investigation was conducted according to the grading standards for potato plants wilt disease. The incidence of plant disease was counted, and the disease index and control effect were calculated using Equations 3 and 4, respectively (Xu et al., 2020).


(3) $$I=100\times \sum \frac{n_{1}\times n_{2}}{n\times 9}$$

where *I* is the disease index, *n_1_* is the number of diseased plants at all levels, *n_2_* is the relative level value, and *n* the total number of plants surveyed.


(4) $$B\left(\%\right)=\frac{I_{1}-I_{2}}{I_{2}}\times 100$$

where, *B* is the biological control effect, *I_1_* is the disease index of the control group, and *I_2_* is the disease index of the treatment group.

### 2.9 Statistical analysis

All genomic analyses were performed according to manufacturer’s instructions. All PGP and antagonistic tests were performed in triplicate. Data were analyzed using analysis of variance and Duncan’s multiple range test.

## 3 Results

### 3.1 Characterization of the antagonistic bacterium Q2H2

**Figure 1:**
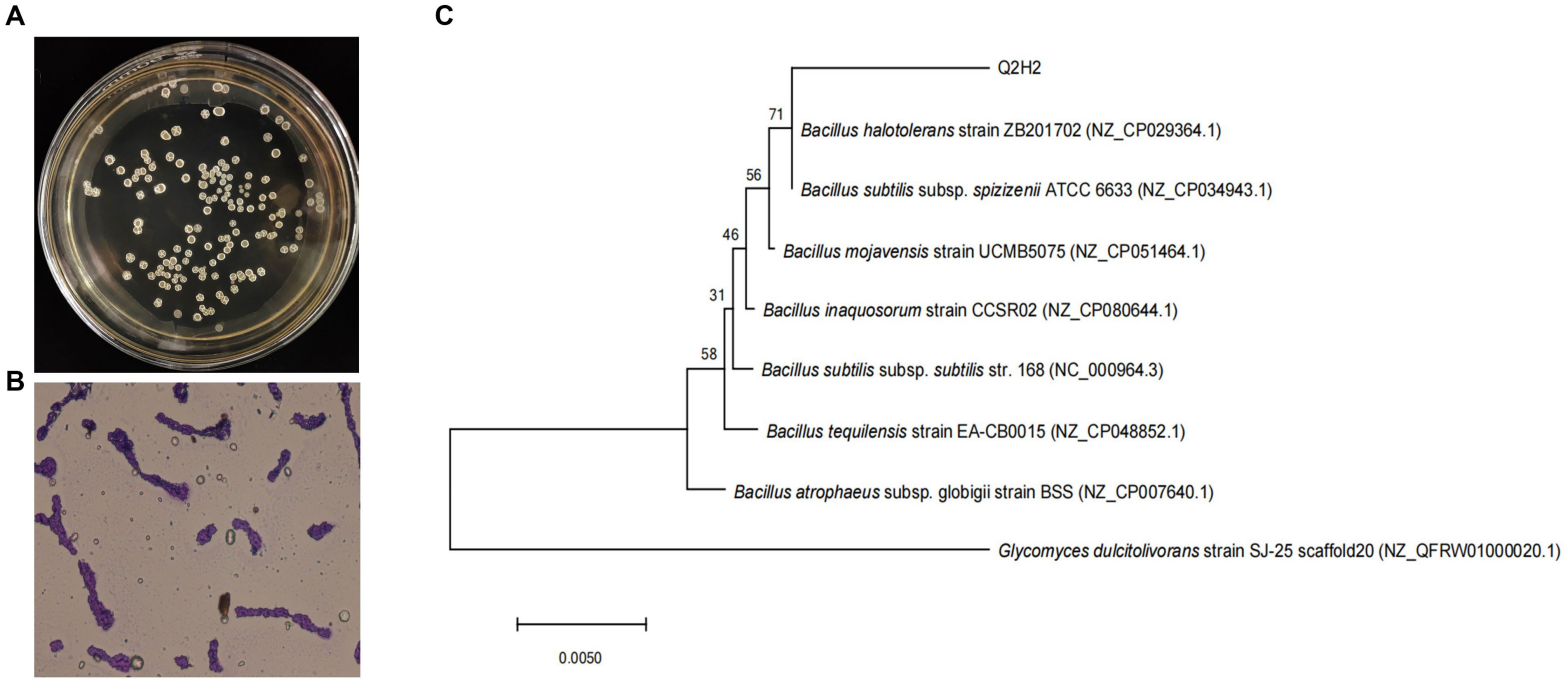
Colony morphological characteristics and phylogenetic analysis of strain Q2H2. **(A)** Colony morphology of bacterial strain Q2H2 on Luria Bertani medium; **(B)** Gram staining of Q2H2; **(C)** Construction of Q2H2 phylogenetic tree based on 16S rRNA gene sequences.

Morphological observations showed that single colonies of Q2H2 were white, flat, opaque, rough, and wrinkled with irregular edges on LB medium (Figure 1A), and Gram staining was positive (Figure 1B). The physiological and biochemical characteristics showed that the Q2H2 catalase enzyme assay, citrate utilization, malonate, methyl red, oxidase, gelatin hydrolysis, starch hydrolysis, and fat hydrolysis tests were all positive. The V-P reactions were negative (Table 2). The partial 16S rRNA gene sequence of Q2H2 was amplified and aligned with 16S rRNA gene sequences deposited in the NCBI database. We constructed a phylogenetic tree with the BLAST results for Q2H2 using MEGA 7.0, and the results are shown in Figure 1C. Q2H2 clustered on the same branch as *B. halotolerans* ZB201702 and *B. subtilis* subsp. *spizizenii* ATCC 6633. Q2H2 was preliminarily identified as *Bacillus* sp.

**Table 2:** Physiological and biochemical characteristics of strain Q2H2.

Physiological and biochemical test	Result	Physiological and biochemical project	Result
Catalase enzyme assay	+	V-P reaction	−
Citrate utilization	+	Hydrolyzed gelatin	+
Malonate utilization	+	Hydrolyzed starch	+
Methyl red test	+	Hydrolyzed fat	+
Oxidase test	+	Gram staining	+

“+”, Positive reaction; “−”, Negative reaction.

### 3.2 Evaluation of antagonistic and PGP activities of strain Q2H2

**Figure 2:**
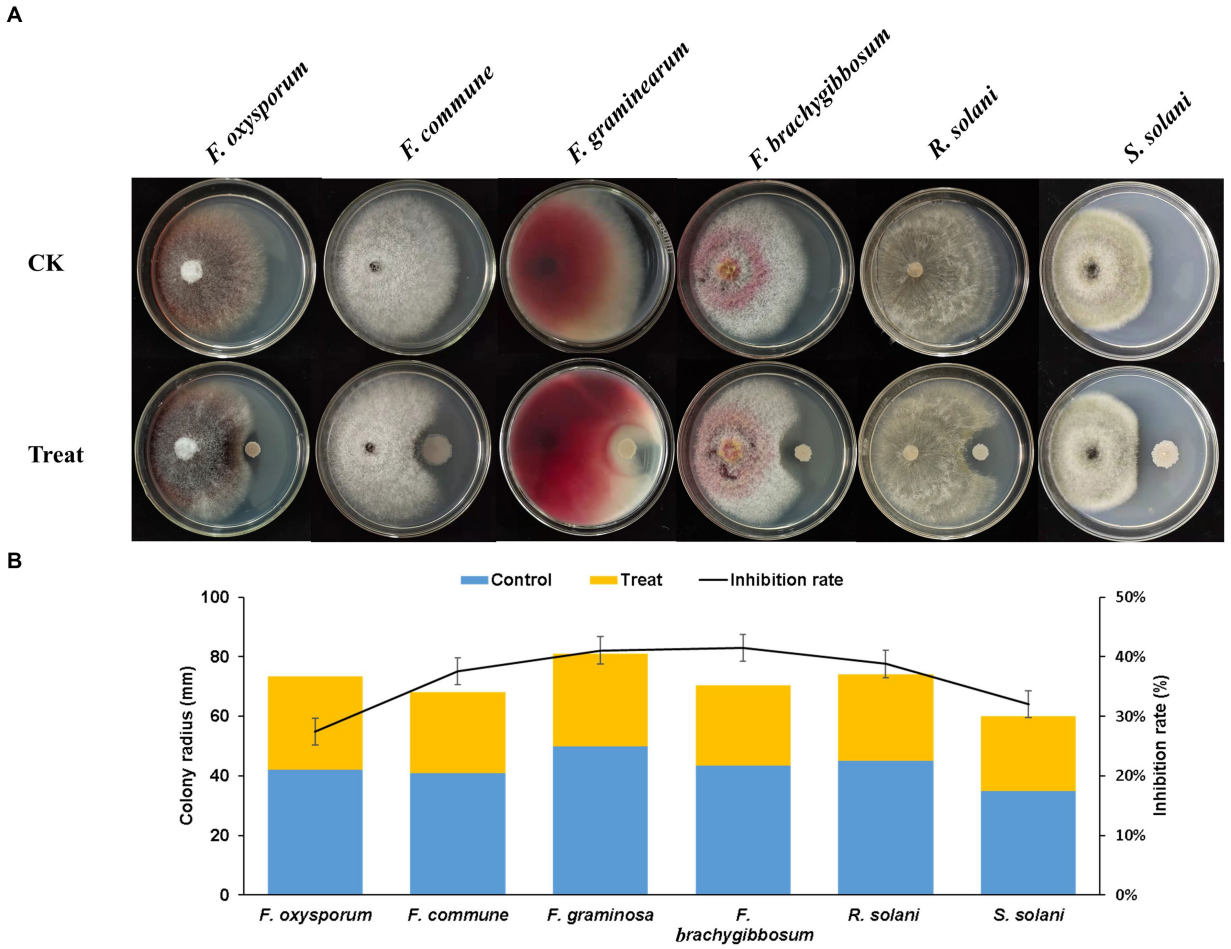
Antagonism of Q2H2 against pathogenic fungi **(A)** Antagonistic effects of Q2H2 against a variety of pathogenic fungi and **(B)** statistical analyses.

The plate antagonism test showed that Q2H2 exerted antagonistic effects against six pathogenic fungi: *F. oxysporum*, *F. commune*, *F. graminearum*, *F. brachygibbosum*, *R. solani*, and *S. solani*. The biocontrol activity results presented in Figure 2A show that the inhibition rate of mycelial growth was highest for *F. brachygibbosum* (41.51%), followed by *F. graminearum* (41.08%). The inhibition rates of *R. solani*, *F. commune, S. solani*, and *F. oxysporum* mycelial growth were 38.79, 37.58, 32, and 27.45%, respectively (Figure 2B).

**Figure 3:**
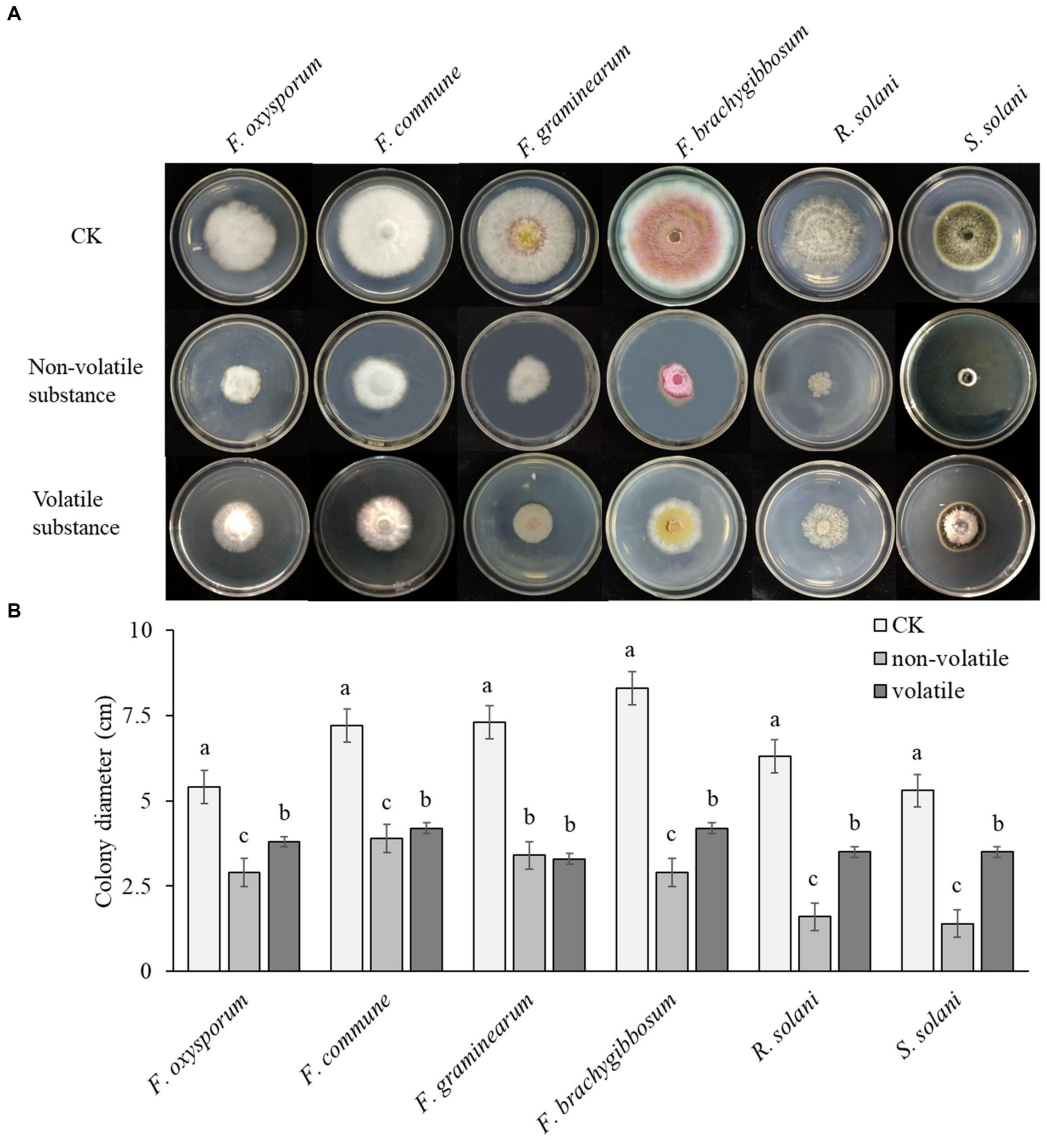
The antibacterial effects of non-volatile and volatile substances of Q2H2 **(A)** Inhibition of non-volatile and volatile substances from Q2H2 on pathogenic fungi; **(B)** Statistical analyses.

The non-volatile antibacterial test showed that, compared to the control, the growth of pathogenic fungi was significantly inhibited on the plate with added fermentation supernatant. The antibacterial rate was calculated by measuring the diameter of the fungal colonies. As shown in Figure 3, Q2H2 had the greatest inhibitory effect against *R. solani* (74.6%), followed by *S. solani* (73.58%). It also had obvious inhibitory effects against *F. oxysporum*, *F. commune*, *F. graminearum*, *F. brachygibbosum*, with antibacterial rates of 46.3, 45.83, 53.42, and 65.06%, respectively. This indicates that the production of certain antibacterial substances in the bacterial fermentation broth can inhibit the growth of pathogenic fungi.

The antibacterial test of Q2H2 volatile substances showed that the most obvious inhibitory effect was against *F. graminearum*, at 54.79%, followed by *F. brachygibbosum* (49.4%). It also showed varying degrees of inhibitory effects against *F. oxysporum*, *F. commune*, *R. solani*, and *S. solani*, reaching 29.63, 41.67, 44.44, and 33.96%, respectively. This indicates that Q2H2 can produce volatile antibacterial substances, but their antibacterial effect is lower than that of the non-volatile substances (Figure 3).

**Figure 4:**
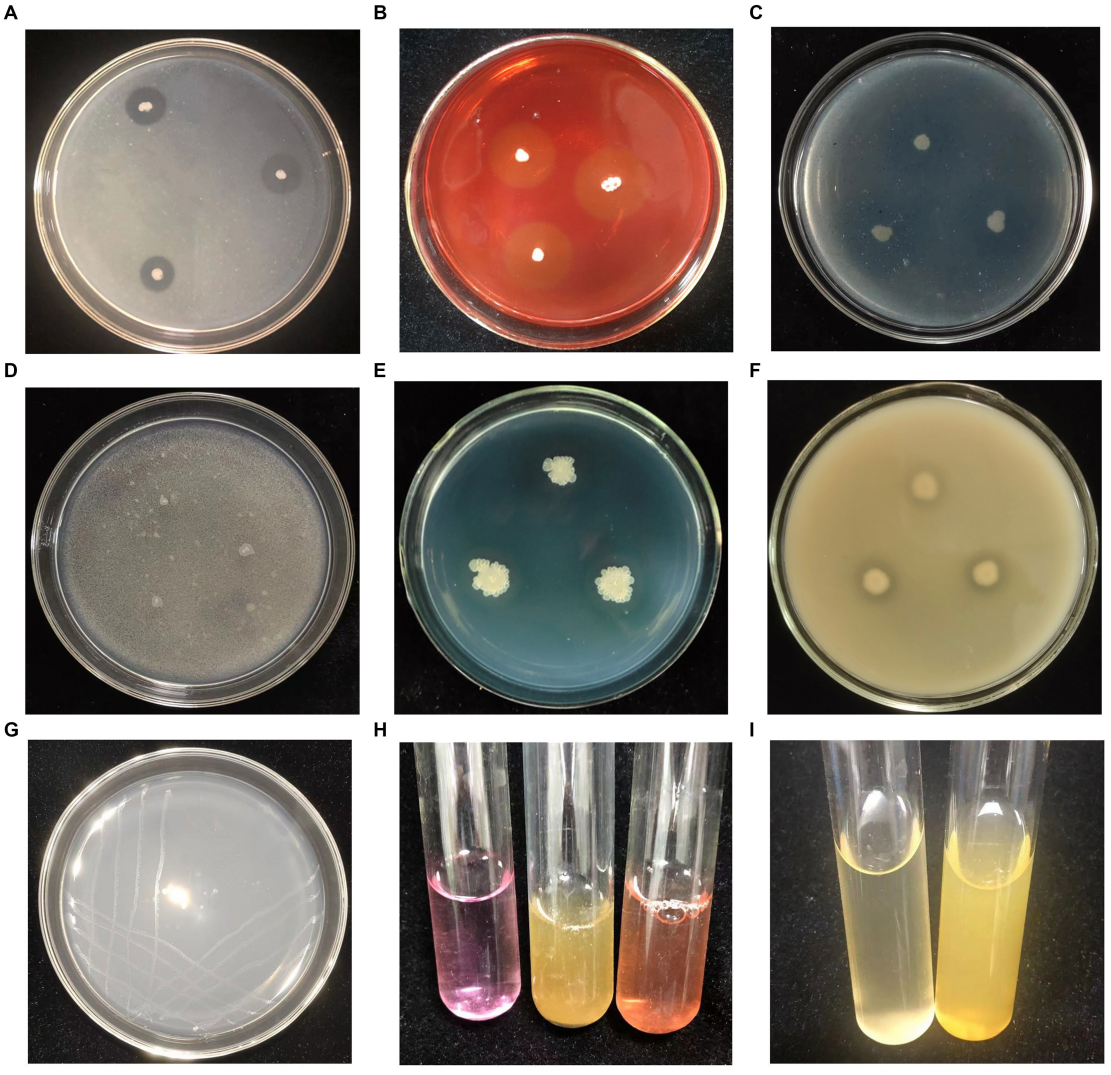
Detection of antagonistic enzymes and plant-growth-promotion activities of Q2H2. **(A)** Protease detection; **(B)** Cellulase detection; **(C)** β-1,3-Glucanase detection; **(D)** Chitinase detection; **(E)** Siderophore detection; **(F)** Organic phosphate detection; **(G)** Nitrogen fixation detection **(H)**; Indole-3-acetic acid (IAA) detection; **(I)** Ammonia detection.

Translucent hydrolysis circles were observed surrounding the Q2H2 colony on the detection medium for protease, cellulase, and β-1,3-glucanase activities, confirming the ability of Q2H2 to produce these enzymes (Figures 4A–C). In addition, Q2H2 grew well on medium with chitin as the sole carbon source; therefore, it was considered to have the ability to produce chitinase, although there was no obvious hydrolysis circle around the Q2H2 colony (Figure 4D).

*In vitro* PGP activity assays of Q2H2 showed that CAS medium produced a brown halo, which confirmed the production of siderophores (Figure 4E). For phosphate solubilization, the Q2H2 culture was dotted onto organic and inorganic phosphate media and grown for 5 days. The results showed obvious halos around the dots in the organic phosphate medium (Figure 4F). Halos were not observed in the inorganic phosphate medium. To test the nitrogen-fixation ability of Q2H2, we performed an assay to grow Q2H2 in medium without nitrogen. We found that Q2H2 grew in the medium without nitrogen (Figure 4G). IAA production was estimated using a colorimetric method in the presence of tryptophan. The mixture of Q2H2 fermentation supernatant and Salkowski reagent showed a clear red color, indicating that Q2H2 can synthesize IAA (31.6 μg/mL) using tryptophan as a precursor (Figure 4H). Q2H2 was inoculated in 10% sterile peptone water at 28°C for 72 h. The color turned yellow upon addition of Nessler’s reagent, confirming the production of ammonia (Figure 4I).

**Figure 5:**
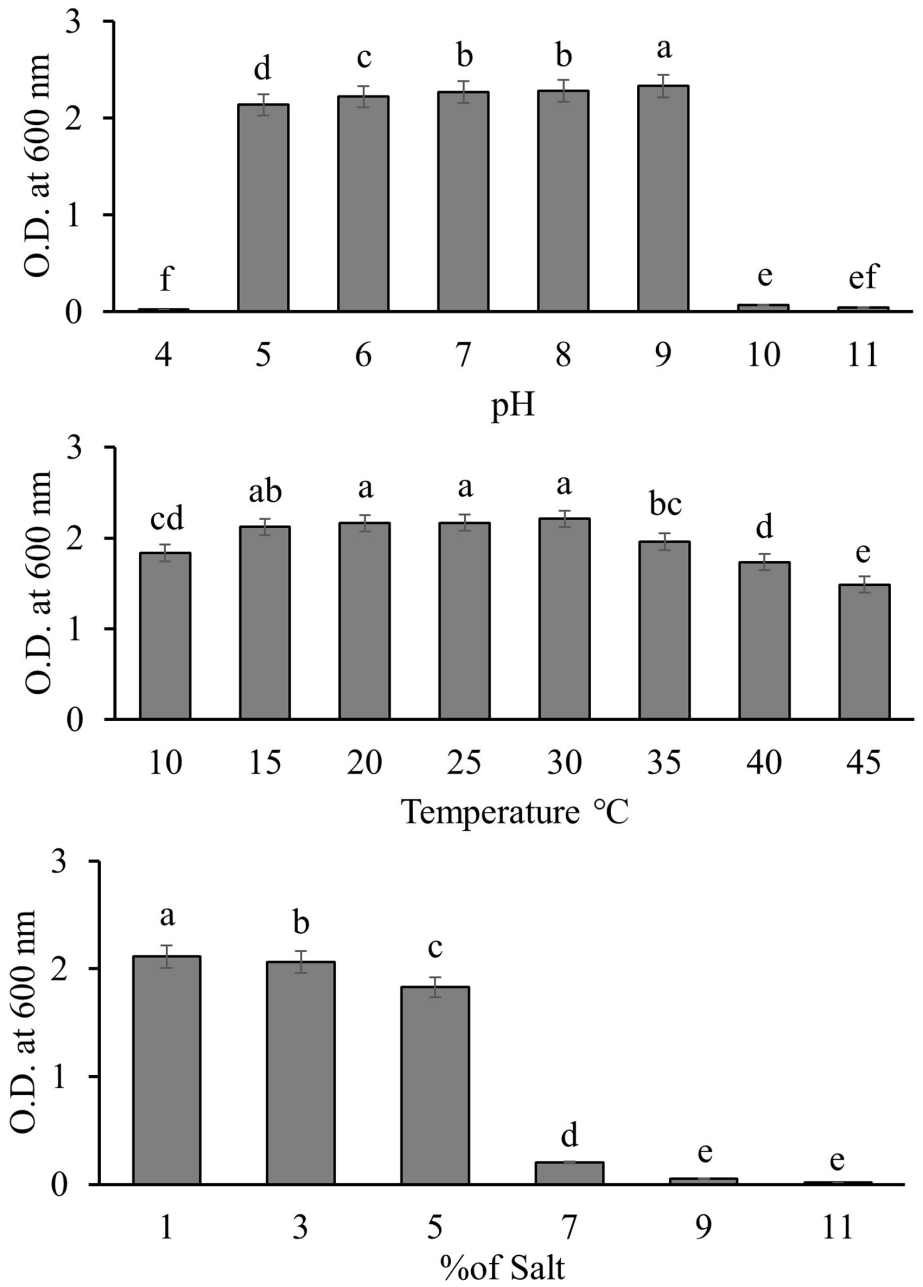
Growth of strain Q2H2 under abiotic stresses such as different pH values (4–11), temperatures (10–45°C), and salt concentrations (1–11%).

We tested the tolerance of Q2H2 under the abiotic stress conditions of pH (4–11), temperature (10–45°C), salt concentration (1–11%) by measuring the absorbance of the bacterial solution at 600 nm. The results are shown in Figure 5. Q2H2 was able to grow normally at pH 5–9. At pH 4, 10, and 11, the growth of Q2H2 was strongly inhibited. At temperatures of 15–30°C, the growth of Q2H2 was not affected, but it was slightly affected at 45°C, indicating that Q2H2 has a strong tolerance to different temperatures. When the salt concentration exceeded 5%, Q2H2 growth was strongly inhibited.

### 3.3 Genomic properties of the endophytic strain Q2H2

**Figure 6:**
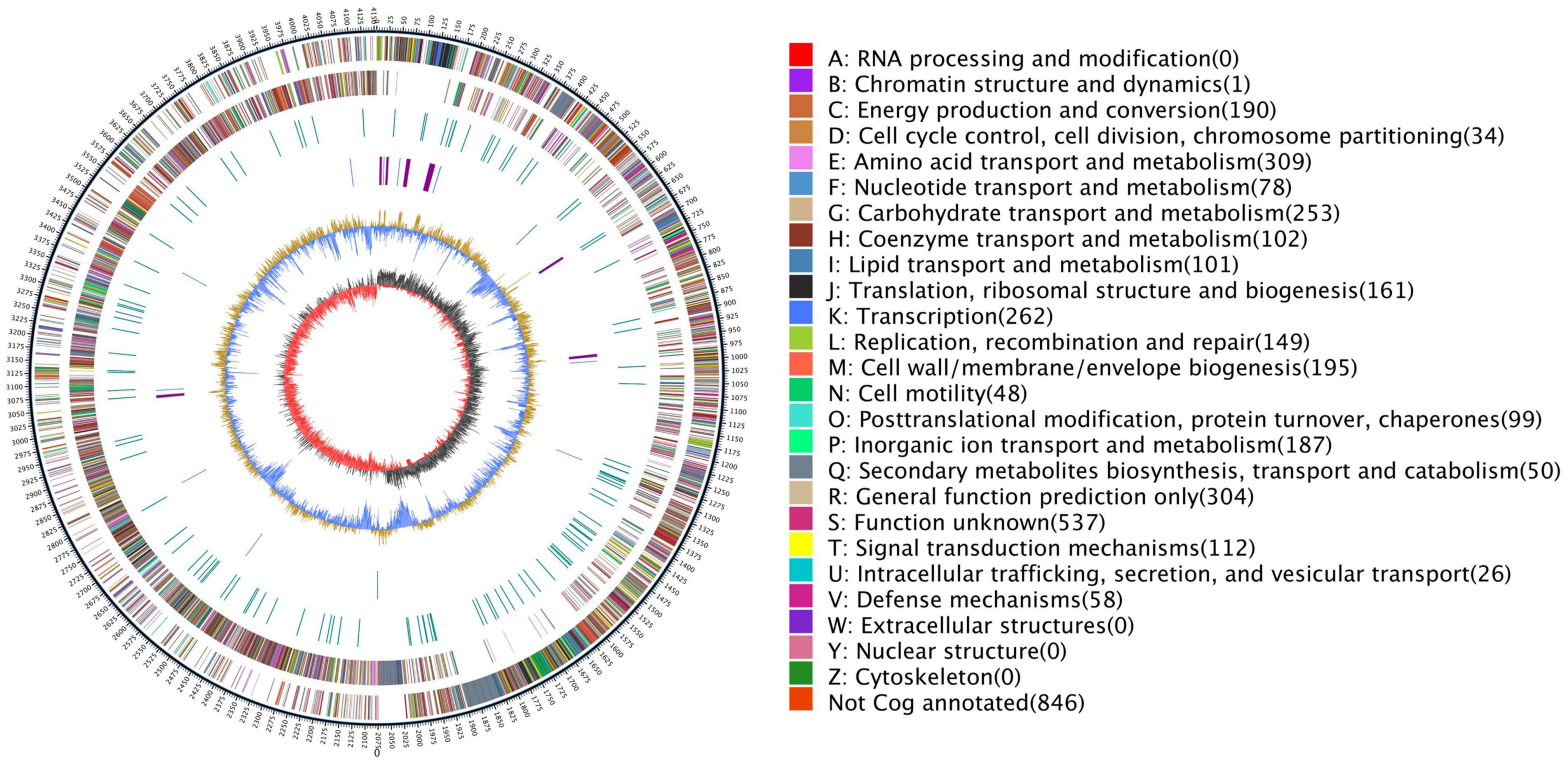
The genome map of Q2H2. The outermost circle indicates the genome size; the second and third circles are genes on the positive and negative strands of the genome, respectively; and different colors represent different Clusters of Orthologous Groups database functional classifications. The fourth circle represents repeat sequences; the fifth circle represents tRNA (blue) and rRNA (purple); the sixth circle represents the GC content; and the innermost circle represents the GC skew. A–Z, respectively, show the functional classifications of the protein-coding genes in the chromosome.

The genome of *B. halotolerans* Q2H2 was found to be composed of a circular chromosome of 4,155,130 base pairs (Table 3) with an overall G + C content of 43.77% and 4,102 protein-coding genes (CDSs), 30 rRNA genes, 86 tRNA genes, and 99 other non-coding RNA genes without a plasmid. Four CRISPR sequences, 18 pseudogenes, 4 gene islands, 4 prophages, 10 gene clusters, 4 promoters, and 4 paralogous genes were predicted. The number of CDSs assigned in the Nr, Gene Ontology (GO), KEGG, eggNOG, Pfam, SwissProt, and TrEMBL databases were 4,062, 3,190, 2,291, 3,255, 3,613, 3,075, and 3,075, respectively. Using the predicted genomic data, a genome map of Q2H2 was created using Circos v0.66 software (Krzywinski et al., 2009). Circos can be used to visualize the genome to explore the relationship between genomic components or locations more clearly (Figure 6). The different colors in Figure 6 represent the different functions of genes annotated in the Clusters of Orthologous Groups database, including amino acid transport metabolism, general function prediction, secondary metabolism biosynthesis, transport, and catabolism. The complete genome sequence of this strain has been submitted to GenBank under the accession number CP136430.

**Table 3:** Genome characteristics of strain Q2H2.

Characteristics	Value
Genome size (bp)	4,155,130
GC content (%)	43.77
Topology	Circular
Chromosome	1
Chromosome size (bp)	4,155,130
Plasmid	0
tRNA	86
rRNA (5S, 16S, 23S)	30
ncRNA	99
Protein-coding genes (CDS)	4,102
Repetitive sequence	4,791 bp (0.12%)
CRISPR	4
Pseudogene	18
Genomic islands	4
Prophage	4
Gene cluster	10
Promoter	4
Paralogous gene	4
Genes assigned to NR	4,062
Genes assigned to GO	3,190
Genes assigned to KEGG	2,291
Genes assigned to eggNOG	3,255
Genes assigned to Pfam	3,613
Genes assigned to Swiss-Prot	3,075
Genes assigned to TrEMBL	3,075

### 3.4 Predictive gene clusters involved in the synthesis of secondary metabolites in Q2H2

To identify the gene clusters involved in the synthesis of secondary metabolites in Q2H2, we used antiSMASH software for prediction and NCBI BLAST comparative analysis of the secondary metabolite gene clusters of Q2H2, as shown in Table 4. The results showed that strain Q2H2 encodes 10 gene clusters involved in the synthesis of secondary metabolites. Among them, four non-ribosomal peptide synthetases (NRPSs), surfactin, bacillaene, fengycin, and bacillibactin; a sactipeptide, subtilosin A; one bacilysin; and four gene clusters encoding unknown products, namely, a type III polyketide synthase (PKS), two terpenes, and a class II lanthipeptide. After comparison with known secondary metabolite gene clusters, the similarity of surfactin was 86%, and the similarity of other products reached 100%. It was determined that Q2H2 can produce these antibacterial substances. This may help us investigate the mechanism of Q2H2 antagonism and anti-microbial peptides. Products with antibacterial effects were identified, including surfactin, bacillaene, fengycin, bacilysin, bacillibactin, and subtilosin A.

**Table 4:** Predictive gene clusters involved in synthesis of secondary metabolites in Q2H2.

Region of genome		Most similar known cluster			
From	To	Type	Productions	Similarity	Resources
361,249	425,159	NRPS	Surfactin	86%	*Bacillus velezensis* FZB42 (Koumoutsi et al., 2004)
1804,142	1910,589	TransAT-PKS, PKS-like, T3PKS, NRPS	Bacillaene	100%	
2,012,760	2,090,080	NRPS, betalactone	Fengycin	100%	
3,752,029	3,793,447	other	Bacilysin	100%	
3,146,063	3,193,202	NRPS	Bacillibactin	100%	*Bacillus subtilis* subsp. *subtilis* str. 168 (Barbe et al., 2009)
3,724,206	3,745,818	Sactipeptide	Subtilosin A	100%	*Bacillus subtilis* subsp. *spizizenii* ATCC 6633 (Stein et al., 2004)
878,043	900,640	Lanthipeptide-class-iii	–		
1,177,318	1,197,771	Terpene	–		
2,160,843	2,182,741	Terpene	–		
2,230,251	2,271,348	T3PKS	–		

### 3.5 Comparative genomics analysis of strain Q2H2

**Figure 7:**
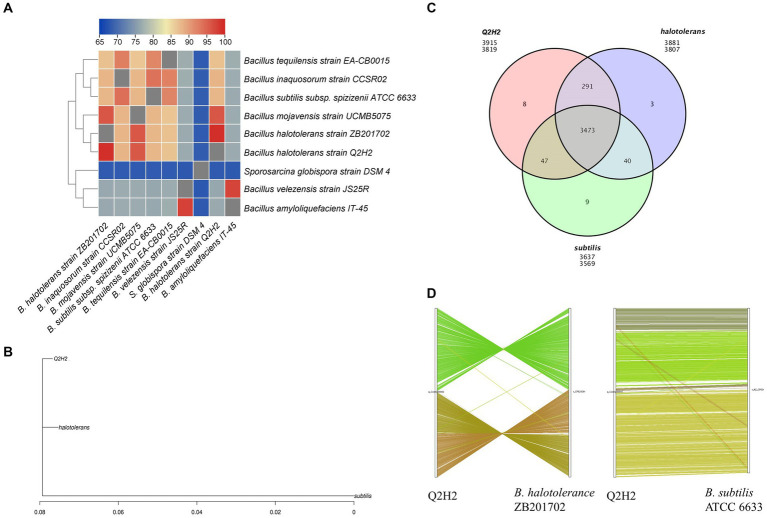
Comparative genomics analysis of strain Q2H2. **(A)** Heat maps of average nucleotide identity (ANI) between strain Q2H2 and eight phylogenetically related species; **(B)** Evolutionary relationships between Q2H2 and two other species; **(C)** Venn diagram of the gene family statistics; **(D)** Genome collinearity of Q2H2/ZB201702 and Q2H2/ATCC 6633.

Heat maps of the ANI between strain Q2H2 and eight phylogenetically related species were created using TBtools software. ANI analysis showed that the similarity was 99.25% between the Q2H2 and *B. halotolerans* strain ZB201702 genomes and 95.88% between the Q2H2 and *Bacillus mojavensis* strain UCMB5075 genomes. The ANI results indicated that Q2H2 was a strain of *B. halotolerans* (Figure 7A).

Analyses of the *16S rRNA* gene sequence and ANI showed that *B. halotolerans* strain ZB201702 was the most similar to Q2H2. Q2H2 was selected as the reference genome for comparative genomic analysis using strains ZB201702 and ATCC 6633. The differential relationships between Q2H2 and the same species and different species were analyzed. An evolutionary tree showing the evolutionary relationships between species was created using PhyML software (Guindon et al., 2010; Figure 7B). These three strains belonged to the same clade, and the evolutionary relationship between Q2H2 and ZB201702 was closer than that of Q2H2 and ATCC 6633. Gene family cluster analysis identified 3,819 genes in the Q2H2 strain, 3,807 in the ZB201702 strain, and 3,569 in the ATCC 6633 strain. The number of gene families shared by the three strains was 3,473, and eight, three, and nine unique genes were detected in the Q2H2, ZB201702, and ATCC 6633 strains (Figure 7C).

The positional and evolutionary relationships of homologous genes on the chromosomes of Q2H2 and related species were explored. The results of the genome collinearity analysis showed that the collinearity of Q2H2 and *B. subtilis* was relatively consistent and only individual gene translocations were observed. However, the collinearity diagram of Q2H2 and ZB201702 shows that there is only a local collinearity region, and a large number of gene inversions and individual gene translocations occurred in the Q2H2 genome (Figure 7D). However, as shown in Figures 7A,B, the evolutionary relationship between Q2H2 and ZB201702 was more similar. We speculate that the difference between Q2H2 and ZB201702 may be due to gene inversion, but the differentiation time was shorter and the accumulation of interspecific variation was less.

### 3.6 Genes efficiently linked with PGP and antagonism in the Q2H2 genome

The PGP characteristics of Q2H2, including phosphate solubilization, nitrogen fixation, siderophore production, IAA production, and ammonia production, were confirmed *in vitro*. To assess antagonistic activity, qualitative tests for cellulase, protease, and chitinase were also performed. We screened for genes related to PGP activity and antagonism in the Q2H2 genome.

Phosphate transporters encoded by *pstB*, *pstA*, *pstC*, and *pstS* were identified in the phosphate metabolic pathway (Supplementary Figure 1). This was consistent with the results of our previous phosphate dissolution experiments. Q2H2 can dissolve organic phosphate, indicating that phosphate transporters in the Q2H2 genome may be closely related to phosphate solubilization (Table 5).

**Table 5:** Predicted genes associated with PGP and antagonistic activities in Q2H2 genome.

Q2H2-PGP activities description	Gene name	Gene annotation	Chromosome location
Phosphate metabolism	*pstB1*	Phosphate import ATP-binding protein PstB 1	2,510,196–2,510,978 (GE002468)
	*pstB2*	Phosphate import ATP-binding protein PstB 2	2,510,989–2,511,798 (GE002469)
	*pstA*	Phosphate transport system permease protein PstA	2,511,819–2,512,703 (GE002470)
	*pstC*	Phosphate transport system permease protein PstC	2,512,703–2,513,632 (GE002471)
	*pstS*	Phosphate-binding protein PstS	2,513,701–2,514,603 (GE002472)
Nitrogen fixation	*nifS*	Putative cysteine desulfurase NifS	2,738,166–2,739,347 (GE002708)
	*nifU*	Nitrogen fixation protein NifU (Fragment)	3,194,492–3,194,728 (GE003142)
	*salA*	Iron–sulfur cluster carrier protein	157,203–158,261 (GE000157)
	*sufU*	Iron–sulfur cluster assembly scaffold protein	3,240,728–3,241,171 (GE003189)
Nitrogen metabolism	*nasE*	Assimilatory nitrite reductase [NAD(P)H] small subunit	356,220–356,540 (GE000331)
	*nasD*	Nitrite reductase [NAD(P)H]	356,572–358,989 (GE000332)
	*gudB*	Cryptic catabolic NAD-specific glutamate dehydrogenase GudB	2,335,422–2,336,696 (GE002265)
	*rocG*	Catabolic NAD-specific glutamate dehydrogenase RocG	3,782,574–3,783,848 (GE003731)
Siderophore	*fhuC*	Iron (3+)-hydroxamate import ATP-binding protein FhuC	3,299,121–3,299,930 (GE003254)
	*fhuG*	Iron (3+)-hydroxamate import system permease protein FhuG	3,299,945–3,300,949 (GE003255)
	*fhuB*	Iron (3+)-hydroxamate import system permease protein FhuB	3,300,949–3,301,998 (GE003256)
	*fhuD*	Iron (3+)-hydroxamate-binding protein FhuD	3,302,203–3,303,150 (GE003257)
IAA Production	*trpA*	Tryptophan synthase alpha chain	2,304,844–2,305,647 (GE002230)
	*trpB*	Tryptophan synthase beta chain	2,305,640–2,306,842 (GE002231)
	*trpF*	N-(5&apos;-phosphoribosyl)anthranilate isomerase	2,306,823–2,307,476 (GE002232)
	*trpC*	Indole-3-glycerol phosphate synthase	2,307,481–2,308,233 (GE002233)
	*trpD*	Anthranilate phosphoribosyltransferase	2,308,226–2,309,242 (GE002234)
	*trpE*	Anthranilate synthase component 1	2,309,214–2,310,761 (GE002235)
	–	Monoamine oxidase	2,117,617–2,119,056 (GE002021)
	*–*	Aldehyde dehydrogenase (NAD+)	–
Hydrolase	*amyE*	Alpha-amylase	324,446–326,446 (GE000303)
	*eglS*	Endoglucanase	2,010,526–2,012,025 (GE001952)
	*gmuD*	Glycoside hydrolase family 1 protein	649,862–651,259 (GE000613)
	*ganB*	Arabinogalactan endo-beta-1,4-galactanase	3,501,710–3,502,279 (GE003347)
Chitinase activity	*sleL*	Cortical fragment-lytic enzyme	23,415–24,698 (GE000017)
	*ydhD*	Putative sporulation-specific glycosylase YdhD	636,940–638,202 (GE000602)
	*-*	Hydrolase, family 18	1,477,988–1,478,923 (GE001448)
	*–*	Hydrolase, family 18	3,386,382–3,387,416 (GE003337)
	*–*	Phytoene/squalene synthase family protein	–
Biofilm	*tasA*	Major biofilm matrix component	2,485,620–2,486,405 (GE002434)
	*bslA*	Biofilm-surface layer protein A	3,072,362–3,072,907 (GE003028)
	*bslB*	Probable biofilm-surface layer protein B	3,784,031–3,784,495 (GE003732)
2,3-butanediol	*alsD*	Alpha-acetolactate decarboxylase	3,597,224–3,597,991 (GE003534)
	*ilvK*	Branched-chain-amino-acid aminotransferase 2	3,859,282–3,860,373 (GE003809)
	*ilvE*	Branched-chain-amino-acid transaminase 1	251,718–252,785 (GE000235)
	*ilvY*	HTH-type transcriptional regulator IlvY	1,515,418–1,516,299 (GE001486)
	*ilvA*	L-threonine dehydratase biosynthetic IlvA	2,226,028–2,227,296 (GE002143)
	*ilvD*	Dihydroxy-acid dehydratase	2,234,047–2,235,723 (GE002153)
	*ilvC*	Ketol-acid reductoisomerase (NADP(+))	2,782,829–2,783,857 (GE002750)
	*ilvH*	Acetolactate synthase small subunit	2,783,881–2,784,399 (GE002751)
	*ilvB*	Acetolactate synthase large subunit	2,784,396–2,786,120 (GE002752)
Methanethiol	*metH*	Methionine synthase	1,206,077–1,207,915 (GE001140)
Isoprene	*ispE*	4-diphosphocytidyl-2-C-methyl-D-erythritol kinase	53,003–53,872 (GE000048)

We also identified a cluster of genes associated with nitrogen fixation in Q2H2 (Table 5). However, we did not identify the complete nitrogenase gene cluster. The nitrogen-fixation-related genes *nifS*, *nifU*, *salA*, *sufU* were annotated in the Q2H2 genome. Two ammonia production pathways were identified in the nitrogen metabolism pathway map of Q2H2 (Supplementary Figure 2). The first includes the enzymes encoded by *gudB* and *rocG*, which catalyze the reduction of L-glutamate to ammonia, and the other includes the enzymes encoded by *nasD* and *nasE*, which catalyze the reduction of nitrite to ammonia. *fhuC, fhuG, fhuB*, and *fhuD* were annotated to encode iron complex transporters, which may be related to siderophores (Table 5).

We identified a series of genes related to IAA production in the Q2H2 genome, which demonstrates the ability of Q2H2 to produce IAA. We identified the tryptophan pathway (TAM pathway) in the KEGG pathway map of tryptophan metabolism, and determined that tryptamine can catalyze the generation of IAA through monoamine oxidase and aldehyde dehydrogenase (NAD+) (Supplementary Figure 3). In addition, a group of tryptophan biosynthesis genes, *trpABFCDE* (Supplementary Figure 4), was found in the tryptophan biosynthesis pathway map and these are closely related to IAA synthesis (Table 5). This is consistent with the *in vitro* IAA determination results for the previously studied strains.

With regard to hydrolase, we annotated the *amyE* and *eglS* genes, which encode α- amylase and endoglucanase. *gmuD* and *ganB* genes were also annotated as cellulases in the Pfam database, which is consistent with the results of *in vitro* experiments showing that Q2H2 has the ability to produce cellulase and glucanase. In the GO database, genes encoding *sleL*, *ydhD*, and hydrolytic enzyme family proteins were associated with chitinase activity (Table 5).

The biofilm synthesis genes *tasA*, *bslA* and *bslB* were also identified, which may be related to the antagonistic activity of Q2H2. The Q2H2 genome predicted some key genes for the synthesis of volatile substances, such as 2,3-butanediol (*alsD* and *ilvABCDEHKY*), methanethiol (*metH*), and isoprene (*ispE*), which may be involved in the biocontrol mechanism of strain Q2H2 (Table 5).

### 3.7 Growth-promoting and biological control effects of potted potato plants under greenhouse conditions

A Q2H2 growth-promotion assay was performed using potato plants grown in a greenhouse. Compared to the control (treated with sterile water), potato plants treated with the Q2H2 bacterial suspension showed a significant increase in the average root weight (2.26 g). Plant height increased by 4.13 cm, fresh weight increased by 1.57 g, chlorophyll content increased by 1.5, nitrogen content increased by 0.47 mg/g, but these differences were not statistically significant compared with the control values (Supplementary Figure 5 and Table 6).

**Table 6:** Statistics of the effect of Q2H2 on potato plants growth.

	Plant height (cm)	Fresh weight (g)	Root weight (g)	Chlorophyll SPAD	Nitrogen (mg/g)
CK	33.50 ± 3.87	41.59 ± 8.26	5.09 ± 1.31	51.38 ± 1.76	18.92 ± 0.56
Q2H2	37.63 ± 6.73	43.16 ± 1,052	7.35 ± 2.23 *	52.88 ± 3.77	19.39 ± 1.21

After 15 days of inoculation of potato plants with *F. oxysporum*, the negative control group showed obvious symptoms of disease, and the disease index value of each treatment was calculated (Supplementary Figure 6). The results of the pot experiment showed that the blank control plant grew healthy. The negative control was only inoculated with *F. oxysporum*, and half of the entire plant showed significant leaf yellowing and wilting, with a severe disease incidence index value of 33.33. After inoculation with strain Q2H2, potato plants inoculated with *F. oxysporum* showed slight yellowing of the lower leaves, with mild symptoms. The disease index value was 16.67, a decrease of 16.66 compared with the control, indicating a significant control effect on *Fusarium* wilt disease, with a control effect of 49.29%. After treatment with carbendazim diluted 1:1500, the lower leaves of the potato began to turn yellow, and there were no wilt symptoms. The disease index was 20, which also showed a clear control effect on Fusarium wilt, reaching 39.14%. Strain Q2H2 had a good control effect on potato *Fusarium* wilt, with a slightly higher effect than that of carbendazim treatment (Table 7).

**Table 7:** Statistics on the control effect of potato Fusarium wilt.

Treat	Disease index	Biocontrol effect (%)
NB	0	−
CK	33.33 ± 3.02 a	−
Q2H2	16.67 ± 2.25 b	49.29 ± 11.41 a
Carbendazim	20.00 ± 2.88 b	39.14 ± 14.21 a

## 4 Discussion

With improvements in low-carbon and sustainable agriculture, biological approaches are urgently needed to minimize crop yield losses resulting from pest activity and to reduce the impact of pest management on human health and the environment (Baker et al., 2020). The biological control of bacteria uses a variety of mechanisms to protect plants from pathogens. One or more mechanisms may be used to prevent or reduce plant diseases and the biological agents used may interact directly or indirectly with pathogens. Biocontrol bacteria can directly interact with pathogens by secreting antibacterial compounds that interfere with their virulence and compete for nutrients and space. Many biocontrol bacteria can synthesize and release metabolites, such as lipopeptides, bacteriocins, antibiotics, biosurfactants, cell-wall-degrading enzymes, and volatile compounds. These metabolites exert antibacterial effects by reducing the growth or metabolic activity of the pathogens (Ku et al., 2021).

An antagonistic endophytic bacterium, Q2H2, was previously isolated in our laboratory at an early stage of infection. In this study, we report the antipathogenic and plant-growth-promoting activities of the functional bacterial strain *B. halotolerans* Q2H2. Additionally, we explored the potential mechanisms underlying the effects of strain Q2H2 using whole-genome sequencing, KEGG pathway enrichment analysis, and the prediction of secondary metabolites.

In the antagonistic activity test of Q2H2, using the plate confrontation method, we found that Q2H2 had a significant inhibitory effect on all six pathogenic fungi tested. In the non-volatile antibacterial test, we added the Q2H2 bacterial fermentation supernatant to the growth medium of pathogenic fungi and found that it had an inhibitory effect on the fungi. Volatile substances also exert inhibitory effects against pathogenic fungi. This indicates that Q2H2 can produce antagonistic secondary metabolites, including non-volatile and volatile organic compounds. Thus, we demonstrated that Q2H2 has good biocontrol potential.

Endophytic bacteria are widely used to promote plant growth. They not only maintain soil fertility, but also reduce the use of chemical fertilizers and improve crop yield (Santoyo et al., 2016). Therefore, this study explored the PGP activities of Q2H2, including phosphate dissolution, nitrogen fixation, siderophore production, IAA production, and ammonia production. The PGP activity test showed nitrogen fixation; dissolved organic phosphate; and the production of ammonia, IAA, proteases, and cellulases. The abiotic stress test showed that the suitable pH range for Q2H2 was 5–9, the suitable temperature range was 15–30°C, and the suitable salt concentration was 1–5%. Pot experiments showed that Q2H2 can promote the growth of potato plant roots and effectively control potato wilt.

Whole-genome sequencing of Q2H2 was performed to explore the possible mechanisms of PGP. Based on the 16S rRNA gene sequencing and ANI analyses, the similarity between Q2H2 and *B. halotolerans* ZB201702 was 99.25%, indicating that Q2H2 is a strain of *B. halotolerans*. Through comparative genome analysis, we found evolutionary relationships that suggest that Q2H2, ZB201702, and ATCC 6633 belong to the same clade (Figure 3B), with Q2H2 and ZB201702 being closer to each other than Q2H2 and ATCC 6633. Gene family cluster analysis of Q2H2, ZB201702, and ATCC 6633 revealed that the three strains shared 3,473 genes, and Q2H2 had eight unique genes. The collinearity between Q2H2 and ATCC 6633 was relatively consistent, and only a few genes were translocated. However, the collinearity diagram of Q2H2 and ZB201702 showed only a local collinearity region, and a large number of inversions and translocations of individual genes has occurred in the Q2H2 genome. We speculate that the differences between Q2H2 and ZB201702 may have been caused by gene inversion; however, the differentiation time is short, and the accumulation of interspecific variation is less.

In the Q2H2 genome, 10 gene clusters involved in the synthesis of secondary metabolites were predicted to be NRPS-type surfactin, bacillaene, fengycin, and bacillibactin; subtilpeptide-type subtilisin A; other types of bacilysin; and four gene clusters encoding unknown products, namely, one type III PKS, two terpenes, and one class III lanthipeptide. Some secondary metabolites, including surfactin, bacillaene, fengycin, bacilysin, bacillibactin, and subtilisin A, have been shown to have antibacterial effects. Bacillibactin has been reported to significantly inhibit the growth and invasion of *Phytophthora capsici* and *F. oxysporum* and effectively reduce disease severity (Woo and Kim, 2008; Yu et al., 2011). According to previous reports, surfactin has broad-spectrum antibacterial activity and significantly inhibits bacterial diseases in plants such as *Arabidopsis* root infection by *Pseudomonas syringae* (Bais et al., 2004) and tomato bacterial wilt caused by *Ralstonia solanacearum* (Xiong et al., 2015). Fengycin, originally discovered in *B. subtilis* F-29-3 in 1986, exhibits antifungal activity against a broad spectrum of filamentous fungi (Vanittanakomt et al., 1986).

Through gene functional annotation, phosphate transporters encoded by *pstB*, *pstA*, *pstC*, and *pstS* were annotated in the Q2H2 genome. We also demonstrated that Q2H2 can dissolve organic phosphates *in vitro*. Similar to our results, other endophytic bacteria, such as *B. subtilis* RS10 (Iqbal et al., 2021), *E. Roggenkampii* ED5 (Guo et al., 2020), and *B. subtilis* EA-CB0575 (Franco-Sierra et al., 2020) have also been reported to dissolve phosphates. A gene related to phosphate transport (*pstACS*) was found in the genome of *B. subtilis* strain RS10 and was confirmed *in vitro* to exhibit good plant-growth-promoting properties. These genes are also present in *E. Roggenkampii* ED5, and have been confirmed *in vitro*. The phosphate-solubilization-related genes *phoU* and *PstABCS* have also been found in the genome of *Peribacillus frigoritolerans* Q2H1 (Wang et al., 2022b).

Only the individual nitrogen fixation-related genes *nifU* and *nifS* were found in Q2H2. The presence of *nifU* and *nifS*, which are required components of the enzymatic module encoding nitrogenase, has previously been demonstrated (Li X. et al., 2016). The *nifU* protein plays a major role in Fe-S cluster aggregation, which is necessary for nitrogen fixation (Smith et al., 2005). This suggests the possibility that strain Q2H2 fixes environmental nitrogen, which was experimentally determined by the growth of *B. halotolerans* Q2H2 on Ashby’s medium. Two ammonia-generation pathways were identified in the nitrogen metabolism pathway diagram of Q2H2. The first type was an enzyme encoded by the *gudB* and *rocG* genes that catalyzes the reduction of L-glutamate to ammonia, whereas the other type was an enzyme encoded by the *nasD* and *nasE* genes that catalyzes the reduction of nitrite to ammonia, which is consistent with the results of our ammonia production experiment. The presence of *nifU*, *glnA*, and *gltBD* in the genome of the sugarcane endophytic bacterium *E. Roggenkampii* ED5 confirms the nitrogen fixation ability of ED5, which significantly promotes the growth of sugarcane (Guo et al., 2020).

When plants grow in iron-deficient environments, siderophores produced by microorganisms can chelate Fe^3+^ ions, which can be difficult for plants to absorb directly for utilization. Siderophores are relatively low molecular weight (500–1,000) organic chelators that bind to insoluble iron in the environment and form Fe^3+^-siderophore complexes. Siderophore-producing bacteria change the availability of iron in the soil through the chelation of siderophores, thereby improving iron availability in the rhizospheres of plants and meeting the nutritional iron needs for plant growth (Garzón-Posse et al., 2019). In this study, we did not find a complete siderophore gene cluster in the Q2H2 genome, but annotated the genes *fhuC*, *fhuG*, *fhuB*, and *fhuD* encoding iron hydroxamate ATP-binding proteins, which may be related to the production of hydroxamate-type siderophores. Consistent with these findings, siderophore production has been detected in *Enterobacter cloacae* SBP8 (Singh et al., 2017) and *Pseudomonas* UW4 (Duan et al., 2013), which have multiple PGP attributes. In addition, we predicted a complete bacillibactin gene cluster in the secondary metabolite gene cluster, and its similarity to the known gene cluster was 100%. The most common siderophore in gram-positive bacteria is catechol bacillibactin, which is produced by *B. subtilis*, *B. cereus*, *B. anthracis*, *B. thuringiensis*, and *B. amyloliquefaciens* (Wilson et al., 2006; Chen et al., 2009). Therefore, bacillibactin in Q2H2 may serve as a siderophore to promote plant health by chelating iron and reducing its absorption by pathogenic fungi.

We identified genes and enzymes involved in tryptophan metabolism in the KEGG metabolic pathway, which is closely related to the synthesis of IAA. A group of tryptophan biosynthesis genes, *trpABFCDE*, was identified in the Q2H2 genome and is closely related to IAA synthesis. Similar to our results, it has been recognized that the presence of tryptophan-related genes in the bacterial genome is related to the production of IAA, such as in *Enterobacter* 638 (Taghavi et al., 2010) and *Enterobacter cloacae* UW5 (Coulson and Patten, 2015). Asaf et al. (2018) identified *trpABD* as a tryptophan biosynthetic gene involved in IAA production in the genome of *Sphingomonas* LK11 (Asaf et al., 2018).

Regarding hydrolases, we annotated the *amyE* and *eglS* genes encoding α-amylase and endoglucanase in the Q2H2 genome. Endoglucanases are members of the cellulase family and play important roles in antagonizing pathogenic fungi. The cellulase-related genes, *gmuD* and *ganB*, were also annotated. The GO database indicates that *sleL* and *ydhD* are related to chitinase activity and may be involved in chitinase synthesis. However, we did not observe any chitinase production ability in *in vitro* chitinase activity measurements, and thus, further research is needed. Chitinase, cellulase, and glucanase genes can damage the cell walls of pathogenic fungi and similar genes have been reported in other strains. The formation of bacterial biofilms can help bacteria effectively colonize plant tissues and is a prerequisite for the biological control of bacterial strains, the inhibition of plant diseases, and the promotion of plant growth. Some strains with PGP activity show antagonistic activity in response to phytopathogens by starting biofilm-like assemblies, as previously reported for *Bacillus cereus* (Xu et al., 2014) and *Paenibacillus* (Timmusk et al., 2015). The biofilm synthesis genes *tasA*, *bslA*, and *bslB* were also identified, which may be related to the antagonistic activity of Q2H2. We annotated key genes encoding volatile substances, such as 2,3-butanediol (*alsD* and *ilvABCDEHKY*), methanethiol (*metH*), and isoprene (*ispE*), which may be involved in the biocontrol mechanism of strain Q2H2. Volatile substances produced by antagonistic bacteria, such as 3-hydroxy-2-butanone (acetoin) and 2,3-butanediol (2,3-butanediol), play important roles in antagonism (Ryu et al., 2003). Volatile organic compounds produced by *Bacillus amyloliquefaciens* BEB17 have an inhibitory rate of approximately 66.86% against the banana wilt pathogen and can inhibit spore germination, causing the mycelia to constrict, expand, and damage the cell membrane (Chen et al., 2018). Some strains with PGP activity, such as *Enterobacter* spp., also produce volatile substances that stimulate plant growth (Weilharter et al., 2011).

## 5 Conclusion

Through PGP and antagonistic experiments, we determined the basic characteristics of the endophytic bacterium Q2H2. We obtained the whole-genome sequence of Q2H2 using genome sequencing. Some genes and gene clusters related to PGP and antagonism were identified, including those involved in phosphate solubilization; nitrogen fixation; and ammonia, siderophore, and IAA production, and hydrolase-related genes. The preliminary mechanisms of growth promotion and antagonism of the endophytic bacterium Q2H2 were also revealed. However, the functional validation of genes related to growth promotion and antagonism has not yet been performed. Further investigations into the functions of these genes will be considered in the future. Our results are of great significance for researching the mechanism of PGP and antagonism and lay a foundation for future plant growth and disease biological control applications.

## Data availability statement

The datasets presented in this study can be found in online repositories. The names of the repository/repositories and accession number(s) can be found at: NCBI – CP136430.

## Author contributions

YW: Writing – original draft. ZS: Writing – original draft. QZ: Writing – original draft. XY: Writing – original draft. YL: Writing – original draft. HZho: Writing – review & editing. MZ: Writing – original draft. HZhe: Writing – review & editing.
