# Supplementary material for: Whole-genome analysis revealed the growth-promoting and biological control mechanism of the endophytic bacterial strain Bacillus halotolerans Q2H2, with strong antagonistic activity in potato plants
Source: Front Microbiol. 2024 Jan 3;14:1287921. doi: 10.3389/fmicb.2023.1287921 (PMC10792059; doi:10.3389/fmicb.2023.1287921)
Supplement: Supplementary file 1 [file Data_Sheet_1.docx]

***Supplementary Material***

**Supplementary Figures**


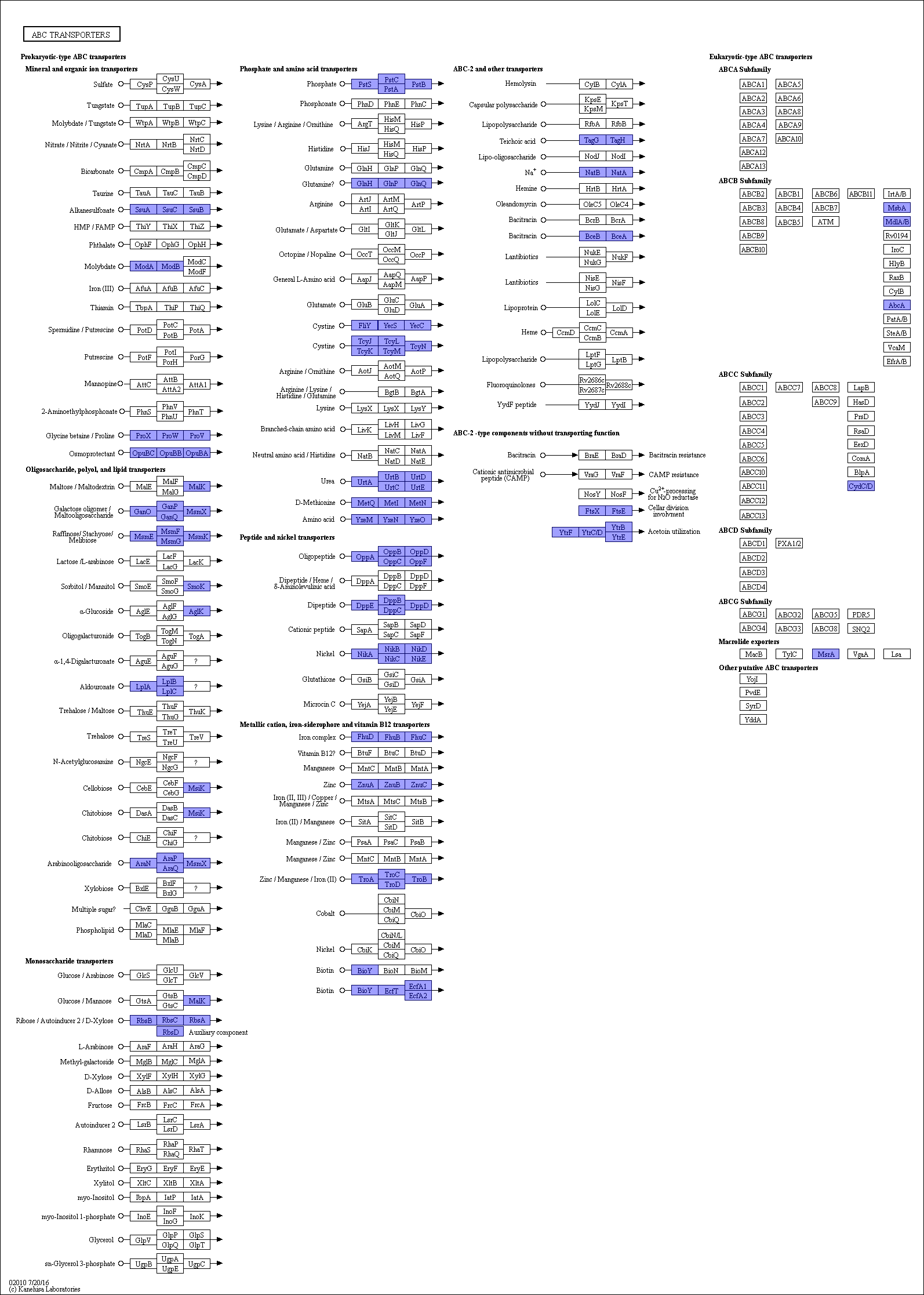


**Supplementary Figure 1.** Q2H2 Phosphate transporters


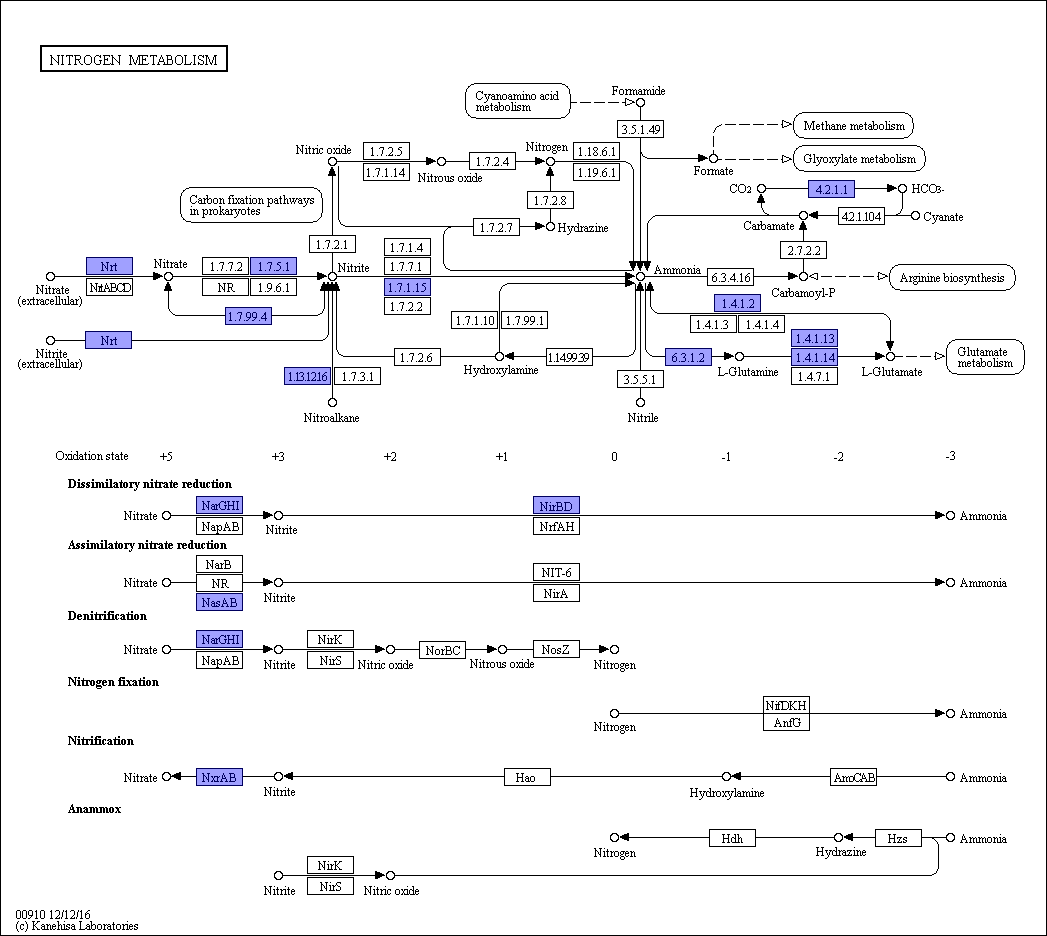


**Supplementary Figure 2.** Q2H2 nitrogen metabolism pathway


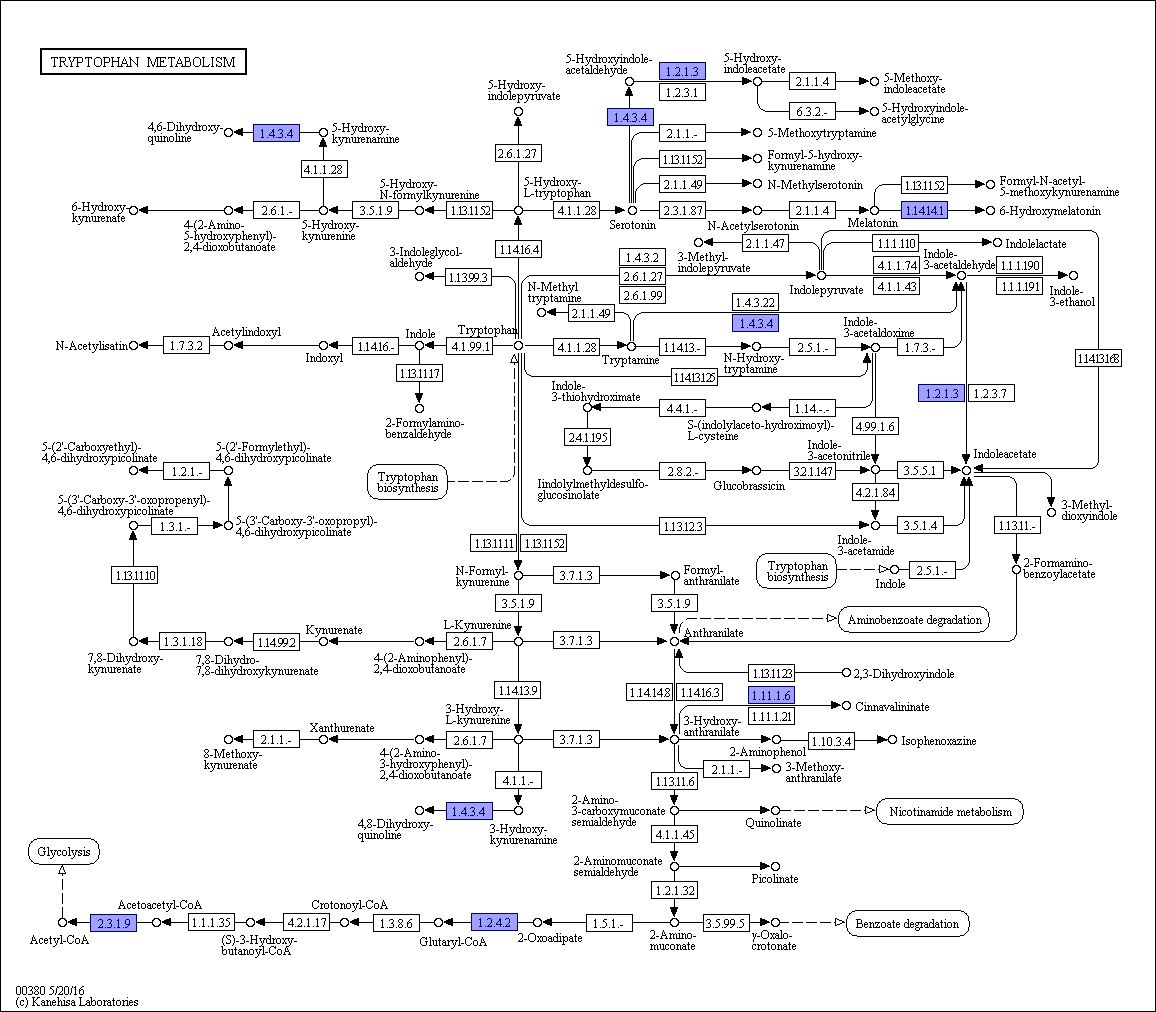


**Supplementary Figure 3.** Q2H2 Tryptophan Metabolism Pathway


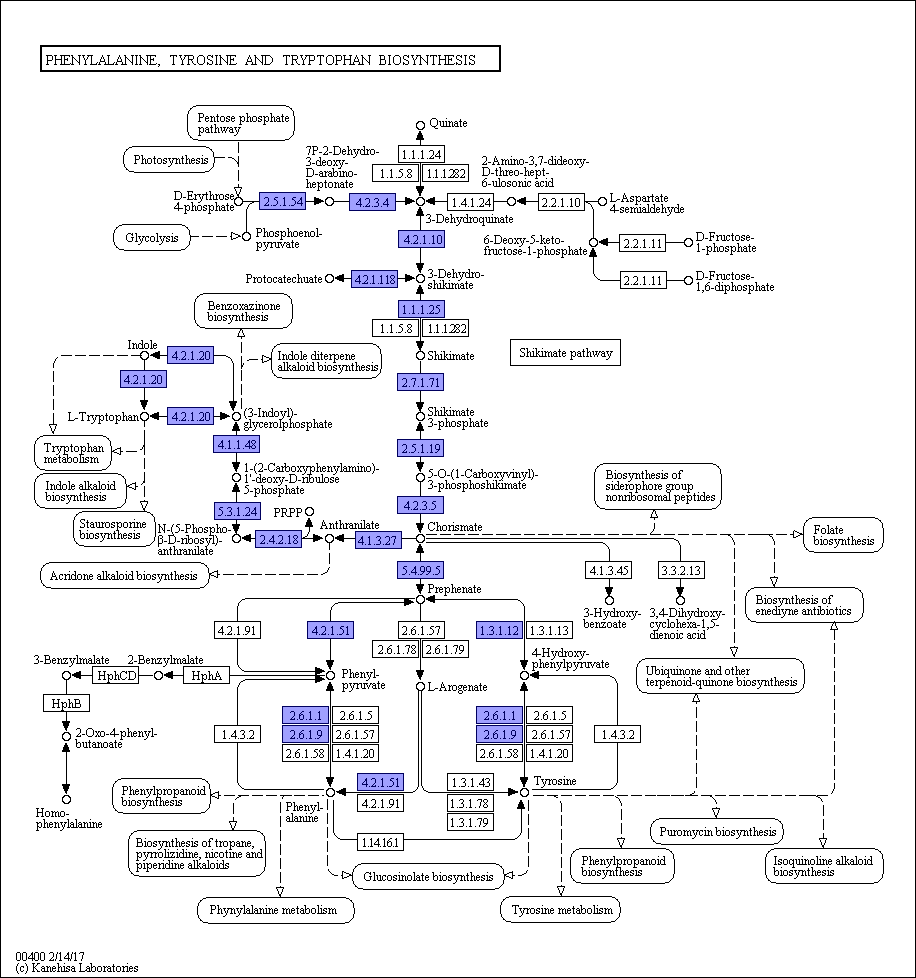


**Supplementary Figure 4.** Q2H2 Tryptophan biosynthesis Pathway


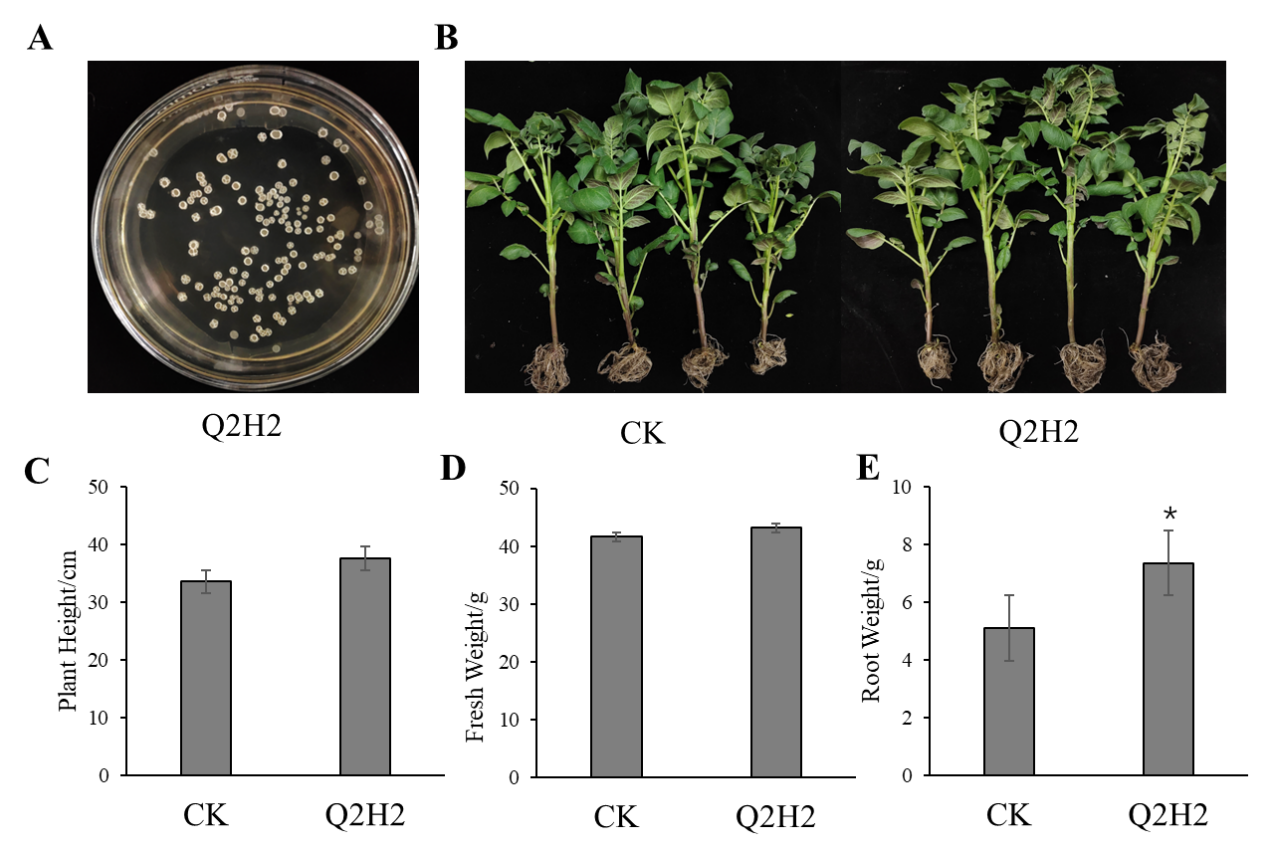


**Supplementary Figure 5.** Plant growth promotion of endophytic strain Q2H2 in potato: **(A)** Colony morphology of strain Q2H2 on LB medium; **(B)** photographs of potato plants at 30 days post-inoculation with Q2H2; **(C, D, E)** plant height, fresh weight and root weight of potato plants measured at 30 days post-inoculation with Q2H2; *show the significant differences (P < 0.05) according to multiple ranges of LSD.


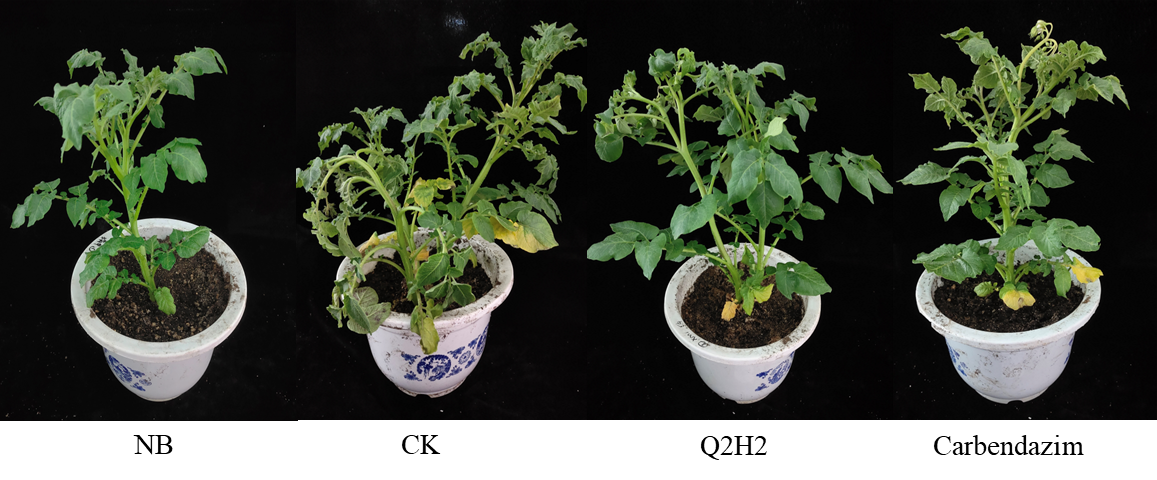


**Supplementary Figure 6.** Pot experiment to verify the control effect of Q2H2 on potato fusarium wilt
